# Pharmacological Blockade of Spinal CXCL3/CXCR2 Signaling by NVP CXCR2 20, a Selective CXCR2 Antagonist, Reduces Neuropathic Pain Following Peripheral Nerve Injury

**DOI:** 10.3389/fimmu.2019.02198

**Published:** 2019-09-26

**Authors:** Anna Piotrowska, Ewelina Rojewska, Katarzyna Pawlik, Grzegorz Kreiner, Agata Ciechanowska, Wioletta Makuch, Irena Nalepa, Joanna Mika

**Affiliations:** ^1^Department of Pain Pharmacology, Maj Institute of Pharmacology, Polish Academy of Sciences, Kraków, Poland; ^2^Department of Brain Biochemistry, Maj Institute of Pharmacology, Polish Academy of Sciences, Kraków, Poland

**Keywords:** CXCL1, CXCL2, glia, microglia, astroglia

## Abstract

Recently, the role of CXCR2 in nociception has been noted. Our studies provide new evidence that the intrathecal administration of its CINC ligands (Cytokine-Induced Neutrophil Chemoattractant; CXCL1-3) induces pain-like behavior in naïve mice, and the effect occurring shortly after administration is associated with the neural location of CXCR2, as confirmed by immunofluorescence. RT-qPCR analysis showed, for the first time, raised levels of spinal CXCR2 after chronic constriction injury (CCI) of the sciatic nerve in rats. Originally, on day 2, we detected escalated levels of the spinal mRNA of all CINCs associated with enhancement of the protein level of CXCL3 lasting until day 7. Intrathecal administration of CXCL3 neutralizing antibody diminished neuropathic pain on day 7 after CCI. Interestingly, CXCL3 is produced in lipopolysaccharide-stimulated microglial, but not astroglial, primary cell cultures. We present the first evidence that chronic intrathecal administrations of the selective CXCR2 antagonist, NVP CXCR2 20, attenuate neuropathic pain symptoms and CXCL3 expression after CCI. Moreover, in naïve mice, this antagonist prevented CXCL3-induced hypersensitivity. However, NVP CXCR2 20 did not diminish glial activation, thus not enhancing morphine/buprenorphine analgesia. These results provide novel insight into the crucial role of CXCR2 in neuropathy based on CXCL3 modulation, which may become a potential therapeutic target in pain treatment.

## Introduction

Neuropathic pain, triggered by peripheral nerve injury, is associated with the plasticity of the nociceptive pathway, where this pain remains, even after the injured tissue has healed (1–5). Mainstream analgesics are not sufficiently successful in achieving selective palliation of neuropathic pain. In fact, these treatments only cause a greater number of side effects. To identify novel alternatives for more effective treatment, it is necessary to clarify the underlying mechanisms. Cytokines, including interleukins and chemokines, are major inflammatory molecules that play an essential role in pain sensitization and have been recently investigated as key mediators in the induction and maintenance of neuropathic pain (6–12).

Chemokines are small cytokines (13), and their participation in neuropathic pain is not limited to their chemotactic activities because these factors also affect the functions of glial and neuronal cells. The evidence of the contribution of chemokines to neuropathic pain includes CX3CL1, CCL2, CCL5, CCL7 CXCL5, CXCL9, CXCL12, and XCL1, and their respective receptors: CCR2, CCR5, CXCR3, CXCR4, and XCR1 (2, 11, 14–28). It has recently been published that a blockade of CCR1 (27), CCR2 (25), CCR5 (28), and CXCR3 (29) restores the analgesic effects of morphine and/or buprenorphine under neuropathy. However, the question of the role of spinal CXCR2 and its endogenous ligands from the CXC (C-X-C motif) family, called cytokine-induced neutrophil chemoattractants (CINCs), belongs to future studies. Among CINCs, three types have been distinguished and are referred to as CINC-1 (chemokine CXC ligand 1, CXCL1; growth-regulated GRO protein alpha, GROα; melanoma growth stimulating activity alpha, MSGA-α; keratinocyte-derived chemokines, KC), CINC-2 (chemokine CXC ligand 3, CXCL3; growth-regulated GRO protein gamma, GROγ; macrophage inflammatory protein-2-beta, MIP2β), and CINC-3 (chemokine CXC ligand 2, CXCL2; growth-regulated protein beta, GROβ; macrophage inflammatory protein 2-alpha, MIP2α). In 2018, Gulati et al. (30) showed that the CINC family arose as a result of two rounds of gene duplication in the course of evolution. The family members are closely related to each other, and biological studies reported their differential tissue expression and regulation. Comparative studies on CXCR2 chemotactic activity have provided evidence of the highest efficacy for CXCL1 and intermediate efficacy for CXCL2 and CXCL3. A previous study showed that all CINCs are expressed by macrophages and play important roles in neutrophil infiltration (31). CINCs act specifically through CXCR2, a G protein-coupled receptor (32, 33), and induce calcium mobilization dose-dependently in CXCR2-transfected cells (34). *In vitro* studies proved that anti-CXCR2 serum almost entirely inhibits the neutrophil chemotactic activities of the three types of CINCs (34).

Therefore, the goal of our studies was to examine the comprehensive roles of all CINCs (CXCL1, CXCL2, and CXCL3) in the pathogenesis of neuropathic pain. Using RT-qPCR and Western blots, we assessed the changes in mRNA expression and protein levels of CXCR2 and its ligands in a rat spinal cord on days 2, 7, 14, and 28 after chronic constriction injury (CCI) of the sciatic nerve. We recognized the origin of CINCs in rat primary cultures of microglia and astroglia by Western blotting. In addition, we made an attempt to visualize the cellular location of CXCR2 and CXCL3 by immunohistochemistry in the lumbar spinal cord on day 7 after CCI. Furthermore, we determined the significance of CXCL1, CXCL2, and CXCL3 in nociceptive transmission in naive mice and the influence of CXCL3 neutralizing antibody in mice on day 7 after CCI. Additionally, another goal of our study involved the determination of how the blockade of CXCR2 signaling through the intrathecal administration of NVP CXCR2 20 affects neuropathic pain-related behavior, glia activation, and the levels of CXCR2 and its endogenous ligands in rats. Eventually, we examined if the CXCR2 antagonist might improve the effectiveness of opioids, such as morphine and buprenorphine in a neuropathic pain model.

## Materials and Methods

### Animals

Adult male Wistar rats (250–300 g) and Albino Swiss mice (20–22 g) from Charles River Laboratories International, Inc. (Germany) were used in our experiments. The rats and mice were housed in cages lined with sawdust under a standard 12/12 h light/dark cycle (lights on at 8.00 a.m.) temperature of 22 ± 2°C with food and water available *ad libitum*. The animals were allowed to acclimate to the environment for ~5 min prior to the behavioral testing. All experiments were performed according to the recommendations of the International Association for the Study of Pain (IASP) by Zimmermann (35) and the National Institutes of Health (NIH) Guide for the Care and Use of Laboratory Animals. The study protocol was approved by the II Local Bioethics Committee branch of the National Ethics Committee for Experiments on Animals based at the Maj Institute of Pharmacology, Polish Academy of Sciences (Krakow, Poland), permission number: 1277/2015 and 262/2017. Care was taken to minimize animal suffering and reduce the number of animals used (3R policy). Animal studies are reported in compliance with the ARRIVE guidelines (36, 37).

### Intrathecal (*i.t*.) Injection

The rats were readied for *i.t*. injection: catheter implants were inserted according to the method described by Yaksh and Rudy (38) and our earlier publications (11, 39, 40). Just before the operation, each rat was anesthetized with sodium pentobarbital (60 mg/kg) administered intraperitoneally (*i.p*.). The *i.t*. catheter included a 13 cm-long polyethylene tubing (PE 10, Intramedic; Clay Adams, Parsippany, NJ, USA). Prior to the insertion for the injection the dead space of 10 μl was sterilized—immersed in 70% (v/v) ethanol and fully flushed with water. Subsequently, 7.8 cm of catheter was introduced through the atlanto-occipital membrane and into the subarachnoid space at the rostral level of the spinal cord lumbar enlargement (L4-L5). The first injection of water (10 μl) was slowly performed after implantation, and the catheter was tightened. All rats recovered after the surgery for 1 week before the establishment of a neuropathic pain model. Repeated *i.t*. drug administration can be achieved due to the catheter implantation. The studies are carried out in a rat model of neuropathic pain, because it allows studying changes in many mediators in one animal at the spinal cord and DRG level in parallel. Regarding the ethical principles of the 3R's, we are obliged to limit the suffering of animals. For this reason, in order to lower the number of animals (rats) subject to a catheter implantation, following the method described by Hylden and Wilcox (41), we performed single drug administrations in mice. Hamilton syringe and a thin needle were used to inject 5 μl of each chemokine between the L5-L6 vertebrae in the spinal cord. The tail reflex indicates the correct drug administration. At the same time, we emphasize that both species of rodents, rats and mice, are commonly used to study the mechanisms of neuropathic pain.

### Neuropathic Pain Model—Chronic Constriction Injury (CCI)

Seven days after the intrathecal catheter insertion in rats, chronic constriction injury to the sciatic nerve was performed according to the method of Bennett and Xie (42). The operation was performed in rats under sodium pentobarbital anesthesia (60 mg/kg; *i.p*.) and in mice under isoflurane anesthesia. An incision was performed under the hipbone, and the separation of biceps femoris and gluteus superficialis. After exposing the proper sciatic nerve, ligatures (4/0 silk) in rats and mice, four and three, respectively, were loosely tied around that nerve at 1-mm intervals until a little twitch in the operated hind limb was obtained. After the surgery, sustained tactile, and thermal hypersensitivity in the injured hind paw developed in each animal.

### Drug Administration

#### All Substances Used in Rats

NVP CXCR2 20 (NVP, Tocris, Janki/Warsaw, Poland), morphine (M; TEVA, Kutno, Poland), and buprenorphine (B; Polfa Warszawa S.A., Warsaw, Poland). NVP CXCR2 20 was dissolved in DMSO, and morphine and buprenorphine were dissolved in water for injections (40, 43, 44). These substances were administered gently through the *i.t*. catheter in a volume of 5 μl, followed by an injection of 10 μl of water, which flushed the catheter. Before the drug injections, the baseline behaviors of the animals were determined using von Frey and cold plate tests. For the single *i.t*. treatment, the behavioral tests were conducted at 0.5, 1, 2, 4, 6 and 24 h after NVP CXCR2 20 injection at a dose of 10, 20, and 30 μg/5 μl. For the repeated *i.t*. treatment, the behavioral tests were carried out 120 (von Frey test) or 125 min (cold plate test) subsequent to NVP CXCR20 20 administration at the selected dose of 10 μg/5 μl according to the following scheme: preemptively at 16 and 1 h following CCI and then once daily for 7 days (28, 40, 45). The dose was chosen based on the results from single *i.t*. treatment behavioral results. For the co-treatment, on the 7th day post-CCI, single V-treated and NVP-treated rats received a single dose of morphine or buprenorphine (2.5 μg/5 μl) at 4 h after the NVP/vehicle injection, and then both behavioral tests were repeated (experimental schedule included in **Figure 9A**). The control groups received vehicle (injection of water or dimethyl sulfoxide, DMSO) according to the same schedule. Our previously study published by Rojewska et al. (46) demonstrated that water for injection- and DMSO-treated CCI-exposed rats developed similarly strong allodynia (11.8 ± 0.4 and 11.9 ± 1.3 g; respectively) and hyperalgesia (6.3 ± 0.5 vs. 6.6 ± 1.6 s; respectively), as demonstrated in the von Frey and cold plate tests. Also in 2016, Rojewska et al. (44) published that 100% DMSO did not influence on hypersensitivity in CCI-exposed rats. In current experiments, an attempt was made to prepare drugs at lower DMSO concentrations, but they precipitate.

#### All Substances Used in Mice

CXCL1, CXCL2, and CXCL3 proteins were obtained from R&D Systems (USA) and dissolved in water for injection. The reconstituted chemokines were intrathecally injected into naive mice at the following concentrations: 2, 400, and 800 ng/5 μl. The behavioral tests were performed at 1.5, 5, and 24 h following the administration of chemokine.

The CXCL3 neutralizing antibody was acquired from R&D Systems (USA) and further dissolved in water for injection. The reconstituted neutralizing antibodies were intrathecally injected into CCI-exposed mice at the following concentrations: 1, 4, and 8 μg/5 μl. The behavioral tests were carried out at 1.5, 5, 24, and 48 h after neutralizing antibody administration.

NVP CXCR2 20 was dissolved in DMSO and intrathecally injected into naive mice at a concentration of 60 μg/5 μl. The behavioral tests were performed at 2 h after CXCR2 antagonist administration. Single V-treated and NVP-treated mice received a single dose of CXCL3 (2 ng/5 μl) at 2 h after the NVP/vehicle injection, and then both behavioral tests were repeated after 1, 5, 5 and 24 h (experimental schedule included in **Figure 7A**).The animals were randomly assigned to groups, based on a single sequence of random assignments, simple randomization—odd/even methods (47, 48).

### Behavioral Tests

#### Tactile Hypersensitivity Measurement (Von Frey Test)

In rats, tactile hypersensitivity was assessed in naive rats and rats subject to CCI with an automated von Frey apparatus (Dynamic Plantar Anesthesiometer, Cat. No. 37400, Ugo Basile, Italy) as previously described (11, 12, 43, 46). Five minutes before the experiment, each rat was placed in a plastic cage with a wire net floor to promote behavioral accommodation. The weight of the von Frey stimuli used in our experiments was up to 26 g. The mid-plantar ipsilateral and contralateral hind paw areas were tested, and the measurements were recorded automatically as described previously (46). No significantly different contralateral hind paw reactions were observed between the CCI and naive rats. Tactile hypersensitivity was assessed at 30 min after the final drug administration.

In mice, the response to non-noxious stimuli was evaluated with von Frey filaments—calibrated nylon monofilaments of increasing strength (from 0.6 to 6 g; Stoelting, USA). The filaments were successively applied to the plantar surfaces of the hind paws until withdrawal responses, as already described (23, 49, 50).

#### Thermal Hypersensitivity Measurement (Cold Plate Test)

In rats, thermal hypersensitivity was determined with a cold/hot plate analgesia meter (Cat. No. 05044/230 VAC, Columbus Instruments, USA) as described previously (11, 12, 43). The rats were placed on a cold stainless steel plate maintained at 5°C, and the latency to lift or shake the injured hind paw was measured. The cut-off latency was 30 s.

In mice, the response to noxious stimuli in the naïve and CCI-exposed animals was evaluated using a cold/hot plate analgesia meter (Cat. No. 35 100-001, Columbus Instruments, USA), as previously described (24, 50). The mice were placed on the cold plate at a temperature of 2°C. The latency of hind paw elevation was recorded. The cut-off latency was 30 s.

### Microglial and Astroglial Cell Cultures

Neonatal models of primary cultures of microglial and astroglial cells were used in our *in vitro* studies as shown previously (11, 12, 51). Both cell types cultures were prepared from 10 1-day-old Wistar rats according to the procedure by Zawadzka and Kaminska (52). The cells were taken from the cerebral cortex and put in poly-L-lysine-coated 75-cm^2^ culture bottles at 3 × 10^5^ cells/cm^2^ density, in high-glucose DMEM with GlutaMAX (Gibco, New York, USA), heat-inactivated 10% fetal bovine serum, 0.1 mg/ml streptomycin, and 100 U/ml penicillin (Gibco, New York, USA). The cultures were maintained at 37°C in 5% CO_2_. On day 4, the medium was changed. On day 9, the cultures were softly shaken and centrifuged to reclaim any loosely adherent microglia. On day 12, the medium was changed, and the microglia were retrieved again. Then, the medium was changed once more, and the cultures were left to grow on a rotary shaker at 37°C for 24 h (200 rpm) to remove the remaining non-adherent cells. The medium was then removed, and the astrocytes were cultured for 3 days and further trypsinized (0.005% trypsin EDTA solution, Sigma-Aldrich, St. Louis, USA). Microglia/astrocytes were seeded in culture medium onto 6-well plates at a final density of 1.2 × 10^6^ cells per well for protein analysis. Primary microglial and astrocyte cell cultures were treated with NVP CXCR20 20 [100 nM] at 30 min before LPS (lipopolysaccharide from *Escherichia coli* 0111:B4; Sigma-Aldrich, St. Louis, USA) administration [100 ng/ml]. The LPS dose was selected basing on the literature (52, 53) and our experience (12, 44, 51). They were then incubated for 24 h for the Western blot analysis (11, 12, 39, 44, 51). We used immunostaining for IBA1 (a microglial marker, SC-327 225, Santa Cruz Biotechnology Inc., Santa Cruz, USA) and GFAP (an astrocyte marker, SC-166 458, Santa Cruz Biotechnology Inc., Santa Cruz, USA) to identify microglia and astrocytes in the cultures. We obtained highly homogeneous microglial and astroglial populations (more than 95% were positive for IBA1 and GFAP, respectively) Zawadzka and Kaminska (52). Only the minimal essential number of animals was used, and all of the procedures were performed according to the recommendations of IASP (35) and the NIH Guide for the Care and Use of Laboratory Animals. The study was carried out in accordance with the recommendations of the local Ethics Committee (Krakow, Poland), permission number: 1277/2015 and 262/2017.

### Biochemical Tests

#### Analysis of Gene Expression by qRT-PCR

Ipsilateral fragments of the dorsal part of the lumbar (L4-L6) spinal cord were collected immediately after decapitation on days 2, 7, 14, and 28 after CCI. Total RNA was extracted with TRIzol reagent (Invitrogen; USA) compliant with the method by Chomczynski and Sacchi (54). A NanoDrop ND-1000 spectrometer (NanoDrop Technologies, Wilmington, USA) measured the RNA concentration in each sample. Reverse transcription was performed at 37°C for 60 min with Omniscript reverse transcriptase (Qiagen Inc., Hilden, Germany) and 1 μg of total RNA from the tissue. The reaction was performed in the presence of an RNAse inhibitor (rRNasin, Promega, Mannheim, Germany) and oligo (dT16) primers (Qiagen Inc., Hilden, Germany). The obtained cDNA templates were diluted 1:10 with H_2_O, and ~50 ng of cDNA templates from each animal were used for each quantitative real-time PCR (RT-qPCR) assay. RT-qPCR was performed with Assay-On-Demand TaqMan probes (Applied Biosystems, Foster City, CA, USA) on an iCycler device (Bio-Rad, Hercules, Warsaw, Poland) in compliance with the manufacturers' protocol. A standard dilution curve established the amplification efficiency in case of each assay. TaqMan primers and probes were used: Rn01527838_g1 (HPRT, hypoxanthine-guanine phosphoribosyltransferase); Rn02130551_s1 (CXCR2, chemokine (C-X-C motif) receptor 2); Rn00578225_m1 (CXCL1, CINC-1, chemokine (C-X-C motif) ligand 1); Rn00586403_m1 (CXCL2, CINC-3, Mip-2, chemokine (C-X-C motif) ligand 2); and Rn01414231_m1 (CXCL3, CINC-2, chemokine (C-X-C motif) ligand 3). A standard dilution curve established the amplification efficiency for each assay (between 1.7 and 2). The cycle threshold values were automatically calculated by CFX Manager v.2.1 software with the default parameters. The RNA content was calculated using the formula 2 (threshold cycle). The level of the HPRT transcript was not significantly changed in the CCI-exposed rats (55), and for this reason it served as an adequate housekeeping gene.

#### Analysis of the Protein Levels (Western Blot)

Ipsilateral fragments of the dorsal part of the lumbar (L4-L6) spinal cord and the DRG (L4-L6 polled into one sample) were collected immediately after decapitation on days 2, 7, 14 and 28 after CCI or at 6 h after the last injection of NVP CXCR2 20 on the 7th day after CCI. The cell lysates (in RIPA buffer with a protease inhibitor cocktail) from primary microglial and astroglial cultures for Western blot analysis were collected at 24 h after LPS stimulation. We collected the lysates from the cell cultures and tissues in RIPA buffer supplemented with a protease inhibitor cocktail. The reaction mixtures were cleared by centrifugation (14,000 × g for 30 min at 4°C). All samples (20 μg of protein from tissue and 10 μg of protein from primary cells) were heated in a loading buffer (4 × Laemmli Buffer, Bio-Rad, Warsaw, Poland) for 8 min at 98°C. Next, the samples were resolved on 4–15% Criterion™ TGX™ precast polyacrylamide gels (Bio-Rad, Warsaw, Poland) and placed on Immune-Blot PVDF membranes (Bio-Rad, Warsaw, Poland) with a semidry transfer (30 min, 25 V). Membranes were blocked with 5% non-fat dry milk (Bio-Rad, Warsaw, Poland) in Tris-buffered saline with 0.1% Tween 20 (TBST) for 1 h at RT, washed with TBST, and incubated with the following commercially available primary antibodies (reactivity of rat and specified by the producer of the observed molecular weight) overnight at 4°C: rabbit anti-Iba-1 (1:1,000, Proteintech, 10904-1-AP), anti-CXCR2 (1:2,000, LSBio, LS-C388292), anti-CXCL1 (1:200, LSBio, LS-C104778), anti-CXCL2 (1:200, Novus, MAB525), anti-CXCL3 (1:250, Novus, AF516), anti-GFAP (1:10,000, Novus, NB300-141), and mouse anti-GAPDH (1:5,000, Millipore, MAB374). Next, the membranes were incubated with 1:5,000 dilutions of horseradish peroxidase-conjugated anti-rabbit or anti-mouse secondary antibodies for 1 h. We used the solutions from a SignalBoost™ Immunoreaction Enhancer Kit (Merck Millipore Darmstadt, Germany) in order to dilute the primary and secondary antibodies. The membranes underwent washing twice with TBST for 2 min each, and 3 times for 5 min each. In the final step, immune complexes were detected with the Clarity™ Western ECL Substrate (Bio-Rad, Warsaw, Poland) and visualized with a Fujifilm LAS-4000 FluorImager system. Fujifilm Multi Gauge software quantified the relative levels of immunoreactive bands. In **Figures 8D–F**, the blots are cropped which was shown with a dotted line on the representation bands below the figures.

#### Immunofluorescence Staining

Immunofluorescent staining was performed on lumbar (L4-L6) spinal cord samples from neuropathic rats on day 7 after CCI. The tissues were fixed in 4% PFA, embedded in paraffin blocks, cut at 7 μm thick slices on a rotary microtome (Leica, Germany), followed by immunofluorescent staining as described by Rafa-Zabłocka et al. (56). After deparaffinization followed by antigen retrieval (microwave method with citrate buffer), the sections were briefly incubated for 30 min in 5% normal pig serum (Vector Labs, USA) in a PBST buffer (0.2% Triton X-100 in phosphate-buffered saline). The sections were incubated overnight at 4°C with the following primary antibodies: anti-CXCR2 (1:100, LSBio, LS-C388292), anti-CXCL3 (1:100, Abcam, ab10064), anti-NeuN (neuronal marker, 1:500, Millipore, MAB377), anti-IBA1 (1:1,000, Abcam, ab139590), and anti-GFAP (1:500, Millipore, AB5541). Antigen-bound primary antibodies were visualized with anti-rabbit Alexa-488-, anti-mouse Alexa-594-, anti-goat Alexa-594-, and anti-chicken Alexa-594–coupled secondary antibodies. The stained sections were assessed and photographed under a fluorescence microscope (Nikon Eclipse 50i, Netherlands). The dorsal part of the lumbar spinal cord was visualized by using representative images of naive and CCI rats. The immunohistochemical study added new information regarding the possible co-localization of CXCR2 and CXCL3 with markers of neurons, micro- and astroglia. These data do not allow quantitative analyses of staining intensity since the experiments were designed to address co-localization only. Factors such as the number of animals per group refrain staining quantitation.

### Statistical Analyses

The number of animals used in the behavioral and biochemical studies was selected based on an earlier study on a similar field (29). All graphs and analyses were prepared using GraphPad Prism 7 software. The data and statistical analysis comply with the recommendations on experimental design and analysis in pharmacology (57).

#### Behavioral Study

The data (**Figures 3A–F**, **4A–D**, **6A,B**, **7B,C**, **9B,C**) are presented in grams and seconds for each group, including the naive groups. The intergroup differences were analyzed via one-way analysis of variance (ANOVA), followed by Bonferroni's test for multiple comparisons. Bartlett's test for homogeneity of variances assessed if the assumption of equal variances was true before employing further statistical tests. Additionally, the results were evaluated using two-way ANOVA to determine the time × drug interaction, if applicable (**Figures 3**, **4**, **6**). In accordance with the 3R rule, the minimum number of animals necessary for conducting statistical analyzes was used in the research.

#### qRT-PCR and Western Blot Studies

**Figure 1:**
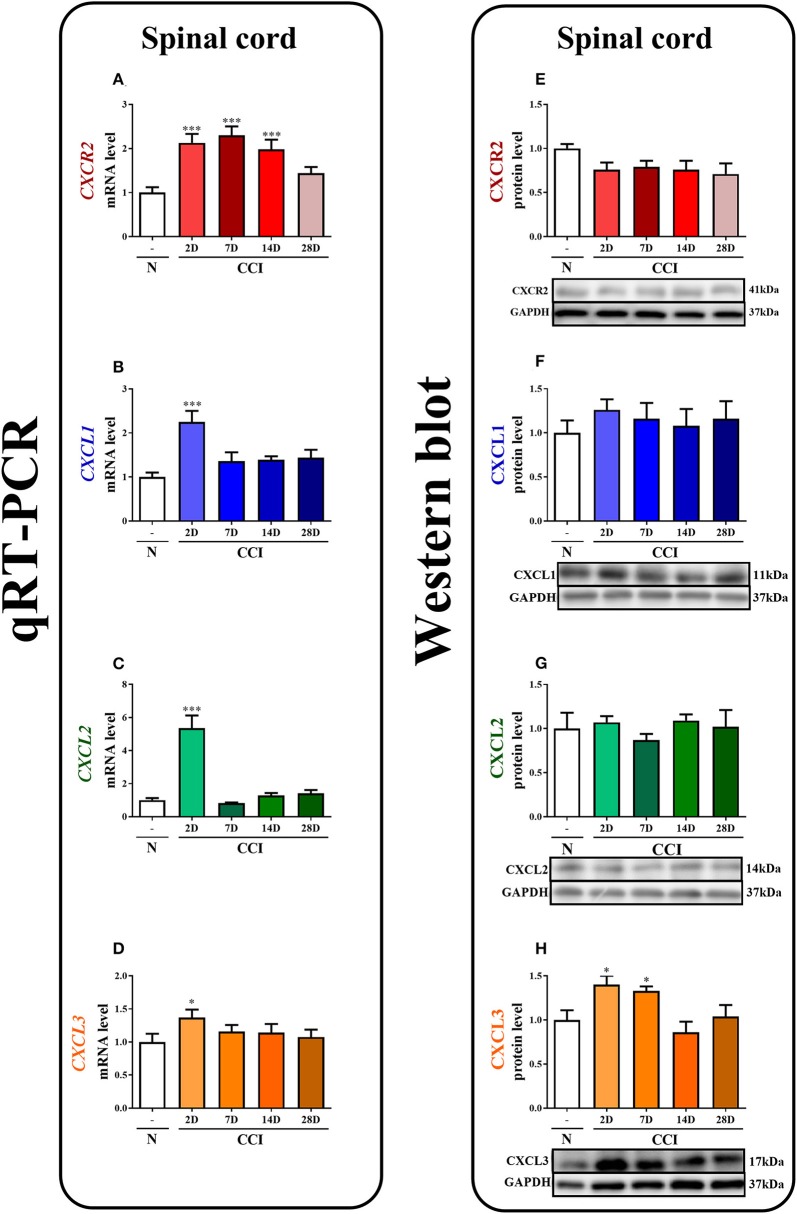
The time course of changes in CXCR2, CXCL1, CXCL2, CXCL3 mRNAs **(A–D)** and proteins **(E–H)** in the spinal cord tissues on the 2nd, 7th, 14th, and 28th days after chronic constriction injury (CCI) in rats. The RT-qPCR and Western blot data are presented as the means ± SEM of 6–10 and 4–6 samples per group in each method, respectively. Intergroup differences were analyzed using ANOVA with Bonferroni's multiple comparisons test. *p < 0.05, ***p < 0.001 indicate differences vs. naive rats. CCI, chronic constriction injury; N, naïve.

*In vivo studies*: The results of the analyses (Figures 1A–H, **5A–I**) are presented as a fold change compared with the control group (naive rats) and were calculated for the ipsilateral side of the spinal cord and/or the DRG on days 2, 7, 14, and/or 28 after CCI or 4/6 h after the last injection of NVP CXCR2 20 on day 7 after CCI. The data are presented as the means ± SEM and represent the normalized averages derived from analyses of each group performed with the Multi Gauge analysis program. Intergroup differences were analyzed using ANOVA, followed by Bonferroni's multiple comparison tests. *In vitro studies:* In case of glial cell cultures, the results of the Western blot analyses (**Figures 8A–F**) are shown as a percentage of the control (vehicle-treated non-stimulated cells) shown as the means ± SEM of 3–4 independent experiments. The results were evaluated with a one-way analysis of variance (ANOVA) with Bonferroni's *post-hoc* test to see the differences between the treated groups. One of the graphs is presented as the relative protein level, and the result was evaluated using a *t*-test to assess the differences between the treatment groups (**Figure 8C**). The variability in the number of samples used in the studies is due to the lack of measurements for technical reasons.

## Results

### The Time Course of Changes in the Levels of CXCR2, CXCL1, CXCL2, and CXCL3 mRNA and Protein in the Spinal Cord on the 2nd, 7th, 14th, and 28th Days After CCI in Rats

In the spinal cord, qRT-PCR analysis showed that the level of CXCR2 mRNA was upregulated 2.1-fold (*p* < 0.001), 2.3-fold (*p* < 0.001), and 2-fold (*p* < 0.001) at 2, 7, and 14 days after CCI, respectively (Figure 1A). The level of CXCL1 mRNA in the spinal cord was significantly enhanced (2.2-fold, *p* < 0.001) only on the 2nd day (Figure 1B). Similarly, the CXCL2 mRNA level was strongly increased (5.4-fold, *p* < 0.001) only on the 2nd day (Figure 1C). The level of CXCL3 mRNA was slightly enhanced (1.4-fold) at 2 days after CCI (Figure 1D).

In the spinal cord, the Western blot analysis showed that no significant changes in the levels of the CXCR2, CXCL1, and CXCL2 proteins were observed post-CCI (Figures 1E–G, respectively). A great increase in the level of CXCL3 protein was detected on the 2nd (1.4-fold, *p* < 0.05) and 7th (1.3-fold, *p* < 0.05) days after CCI (Figure 1H; Data Sheet 1).

### The Spinal Localization of CXCR2 and Its Ligand CXCL3 on the 7th Day After CCI in Rats

**Figure 2:**
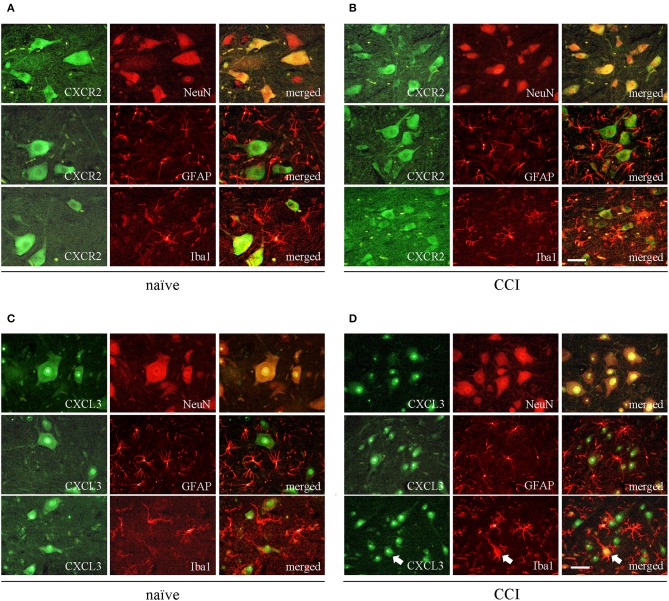
The spinal localization of CXCR2 and its ligand CXCL3 in naive and CCI-exposed rats. Immunofluorescent staining was performed on paraffin-embedded 7 μm **(A,B)** co-staining of CXCR2 (green) and neuronal marker NeuN (red; upper row); astroglial marker GFAP (red, middle row), and microglial marker IBA1 (red, bottom row). **(C,D)** co-staining of CXCL3 (green) and neuronal marker NeuN (red; upper row); astroglial marker GFAP (red, middle row), and microglial marker IBA1 (red, bottom row). White arrows indicate representative CXCL3-positive cells that co-localize with IBA1-positive cells. Scale bar for all pictures: 25 μm.

The immunofluorescent staining provided clear evidence that both CXCR2 and CXCL3, regardless of treatment (naive vs. CCI) co-localize with neurons, as shown by double staining using the neuronal marker, NeuN (Figures 2A–D, upper rows). Co-staining with microglia marker, IBA1, and astroglia marker, GFAP, showed lack of co-localization of CXCR2 or CXCL3 with Iba1 or GFAP (Figures 2A–D, middle and bottom rows), however in CCI-induced animals there were possible to find a few, singular cells co-stained with CXCL3 and IBA1 (Figure 2D, indicated by arrows), revealing that at least under enhanced inflammatory response some CXCL3-positive cells may also expressed activated microglia.

### The Influence of the Single Intrathecal Administration of CXCL1, CXCL2, and CXCL3 on Nociceptive Transmission in Naive Mice

**Figure 3:**
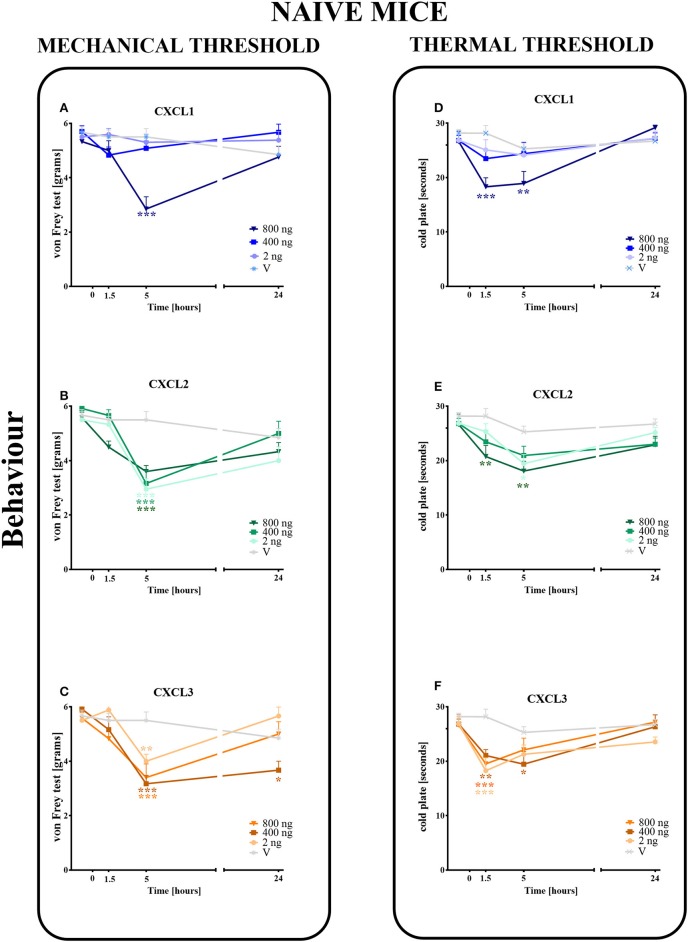
Effects of single administrations of CXCL1, CXCL2, and CXCL3 **(A–F)** on nociceptive transmission in naive mice. The effects of single intrathecal administrations of CXCL1, CXCL2, CXCL3 (2, 400, or 800 ng/5 μl) on mechanical hypersensitivity (von Frey test, **A–C**) and thermal hypersensitivity (cold plate test, **D–F**) were measured at 1.5, 5, and 24 h after administration. Data are presented as the means ± SEM (6 mice per group). The results were evaluated using one-way ANOVA, followed by Bonferroni's test for comparisons of selected pairs measured separately at each time point. *p < 0.05, **p < 0.01, ***p < 0.001 for the comparison of vehicle-treated naive animals with all groups at the indicated time points. Additionally, the results were evaluated using two-way ANOVA to determine the time × drug interaction (please see results in Chapter 3.3). V, vehicle.

The single intrathecal administration of different doses of CXCL1, CXCL2, and CXCL3 induced the development of mechanical and thermal hypersensitivity, as measured using the von Frey (Figures 3A–C) and cold plate (Figures 3D–F) tests, respectively.

In the von Frey test, no significant pronociceptive effects were observed after a low dose of CXCL1 (2 ng/5 μl) at all studied time points (1.5–24 h). However, the high doses (400 and 800 ng) evoked mechanical hyperalgesia 1.5 h (*p* < 0.001) or 5 h (*p* < 0.001) after injection (Figure 3A). This effect vanished after 24 h. For CXCL2 and CXCL3, no significant reactions were observed at 1.5 h after the injection of all doses (2, 400, and 800 ng). However, at 5 h after the injection of CXCL2, the effects of all doses were strong (*p* < 0.001), which nevertheless disappeared altogether at 24 h (Figure 3B). Similarly, the pronociceptive effect of CXCL3 appeared later, and the strongest mechanical hypersensitivity was observed for all doses after 5 h (*p* < 0.001 for 400 ng; *p* < 0.01 for 800 ng; *p* < 0.01 for 2 ng). The effect faded after 24 h, except for one dose (400 ng) (Figure 3C). Two-way ANOVA confirmed a significant interaction [*F*_(9, 110)_ = 5.783, *p* < 0.0001; *F*_(9, 104)_ = 2.689, *p* = 0.0075; *F*_(9, 104)_ = 3.331, *p* = 0.0013; respectively] between the investigated treatments for CXCL1, CXCL2, CXCL3, and the investigated time points. CXCL1, CXCL2, and CXCL3 significantly decreased the nociceptive threshold [*F*_(3, 110)_ = 6.268, *p* = 0.0006; *F*_(3, 104)_ = 26.87, *p* < 0.0001; *F*_(3, 104)_ = 20.32, *p* < 0.0001], showing a pronociceptive dose-dependent effect of CXCL1, CXCL2, and CXCL3 in the von Frey test.

In the cold plate test, no significant pronociceptive effects were observed after low (2 ng/5 μl) and intermediate (400 ng/5 μl) doses of CXCL1 (Figure 3D) and after an intermediate (400 ng/5 μl) dose of CXCL2 (Figure 3E) at all studied time points (1.5–24 h). The mice displayed thermal hypersensitivity to stimuli at 1.5 h (*p* < 0.001) and 5 h (*p* < 0.01) after the injection of the highest dose of CXCL1 (800 ng), which nevertheless disappeared until 24 h (Figure 3D). Similarly, at 1.5 h (*p* < 0.01) and 5 h (*p* < 0.01) after CXCL2 injection, we observed the highest peak reaction for the highest dose and in addition to the low dose after 5 h (*p* < 0.05) (Figure 3E). All tested doses of the CXCL3 injection caused comparable reactions to thermal stimuli after 1.5 h (*p* < 0.001 for 2 and 800 ng; *p* < 0.01 for 400 ng). Only the reaction after the intermediate dose was also maintained after 5 h (*p* < 0.01) (Figure 3F). Two-way ANOVA confirmed a significant interaction [*F*_(9, 124)_ = 2.605, *p* = 0.0087] between the investigated treatment for CXCL1 and the investigated time points. In the case of CXCL2 and CXCL3, two-way ANOVA did not show a time × drug interaction [*F*_(9, 120)_ = 0.8918, *p* = 0.5349; *F*_(9, 118)_ = 1.905, *p* = 0.0577, respectively]. CXCL1, CXCL2, and CXCL3 significantly decreased the nociceptive threshold [*F*_(3, 124)_ = 2.605, *p* = 0.0087; *F*_(3, 120)_ = 9.048, *p* < 0.0001; *F*_(3, 118)_ = 10.31, *p* < 0.0001, respectively], showing a pronociceptive effect of CXCL1, CXCL2, and CXCL3 in the cold plate test.

### Effect of the Single and Repeated Intrathecal Administrations of the CXCR2 Antagonist NVP CXCR2 20 on Mechanical and Thermal Hypersensitivity in the CCI-Induced Model of Neuropathic Pain

**Figure 4:**
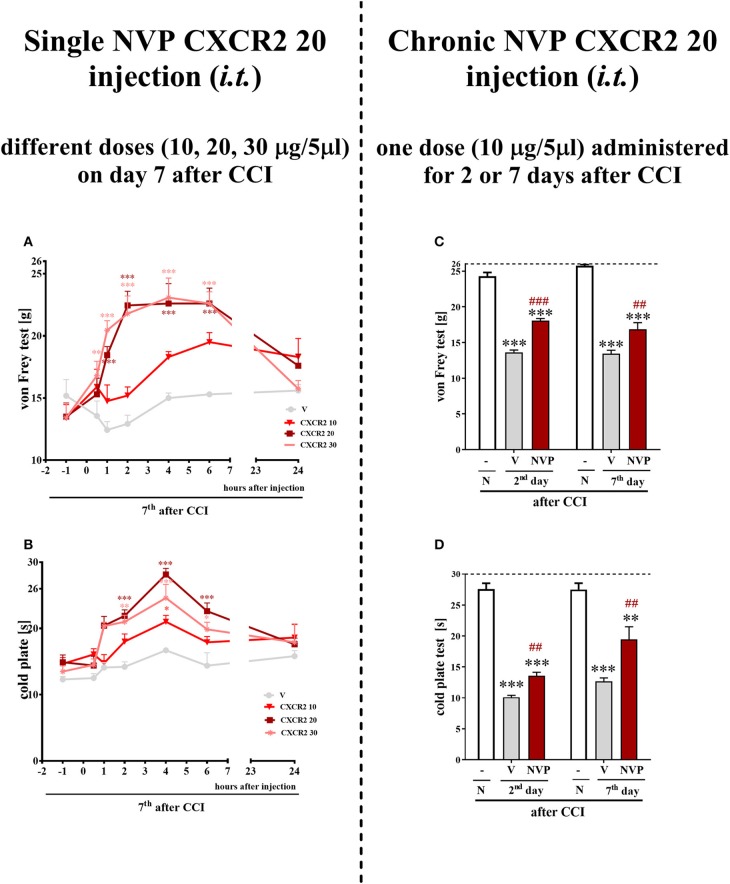
Effects of single **(A,B)** (different doses: 10, 20, and 30 μg/5 μl) intrathecal NVP CXCR2 20 administration on mechanical (**A**; von Frey test) and thermal (**B**; cold plate test) hypersensitivity as measured 0.5, 1, 2, 4, 6, 24 h after NVP CXCR2 20 injection on day 7 in CCI-exposed rats. Effects of repeated **(C,D)** (one dose: 10 μg/5 μl *i.t*.; 16 h and 1 h before CCI and then once a day for 7 days) intrathecal NVP CXCR2 20 administration on mechanical (**C**; von Frey test) and thermal (**D**; cold plate test) hypersensitivity as on day 2 or 7 in CCI-exposed rats. Tactile and thermal hypersensitivity were assessed at 120 and 125 min after the last NVP CXCR2 20 injection, respectively. The horizontal dotted line shows the cut-off value. Data are presented as the means ± SEM of 10–18 rats after single administration and 8 rats after repeated administration per group. Intergroup differences were analyzed using ANOVA with Bonferroni's multiple comparisons test measured separately at each time point. *p < 0.05, **p < 0.01, ***p < 0.001 indicate differences between vs. naive rats. ^*##*^*p* < 0.01, ^*###*^*p* < 0.001 indicate differences between V- and NVP-treated, CCI-exposed rats. CCI, chronic constriction injury; N, naive; V, vehicle; NVP, NVP CXCR2 20. Additionally, the results presented on graphs A and B were additionally evaluated using two-way ANOVA to determine the time × drug interaction (please see results in chapter 3.4).

A single *i.t*. administration of NVP CXCR2 20 at concentrations of 10, 20 and 30 μg/5 μl was performed at 7 days after CCI. The influence of the NVP CXCR2 20 on the development of hypersensitivity to mechanical and thermal stimuli was measured by von Frey (Figure 4A) and cold plate (Figure 4B) tests, respectively, at 0.5, 1, 2, 4, 6, and 24 h after administration. NVP CXCR2 20 injection at the lowest dose did not diminish mechanical hypersensitivity (Figure 4A) and only slightly diminished thermal hypersensitivity (Figure 4B). However, higher doses showed significant analgesic effects at 2, 4 and 6 h after injection measurement by cold plate and von Frey tests (Figures 4A,B). Two-way ANOVA confirmed a significant interaction [*F*_(18, 110)_ = 4.054, *P* < 0.0001; *F*_(18, 133)_ = 2.716, *P* = 0.0006, respectively] between the investigated treatment and the investigated time points in von Frey and cold plate tests. The mechanical and thermal hypersensitivity were significantly diminished after NVP CXCR2 20 treatment [*F*_(6, 110)_ = 16.29; *P* < 0.0001; *F*_(6, 133)_ = 25.58; *P* < 0.0001; respectively]. Based on the obtained behavioral results and our pharmacological experience for the repeated treatment, we have chosen the dose of 10 μg/5 μl, so the lowest possible dose with analgesic effect to avoid side effects.

Repeated *i.t*. administration of NVP CXCR2 20 at a concentration of 10 μg/5 μl has analgesic effects in CCI-treated rats (Figures 4C,D). After CCI, all rats exhibited strong mechanical hypersensitivity in the paw ipsilateral to the injury (as demonstrated by the von Frey test results on days 2 and 7 after CCI (*p* < 0.001) (Figure 4C), and compared to the control group of naive animals, all rats exhibited potent thermal hypersensitivity (as demonstrated by the response latency in the cold plate test (*p* < 0.001; Figure 4D). NVP CXCR2 20 reduced mechanical (*p* < 0.001) (Figure 4C) and thermal (*p* < 0.01) hypersensitivity at 120 and 125 min after the last injection on day 2 after CCI (Figure 4D). On day 7 after CCI, NVP CXCR2 20 also diminished mechanical (*p* < 0.01) (Figure 4C) and thermal (*p* < 0.01) hypersensitivity (Figure 4D) at 125 min after the last injection.

### The Influence of the Repeated Administration of NVP CXCR2 20 on CXCR2, IBA1, GFAP, CXCL1, CXCL2, and CXCL3 Protein Levels in the Spinal Cord and DRG at 7 Days After CCI in Rats

**Figure 5:**
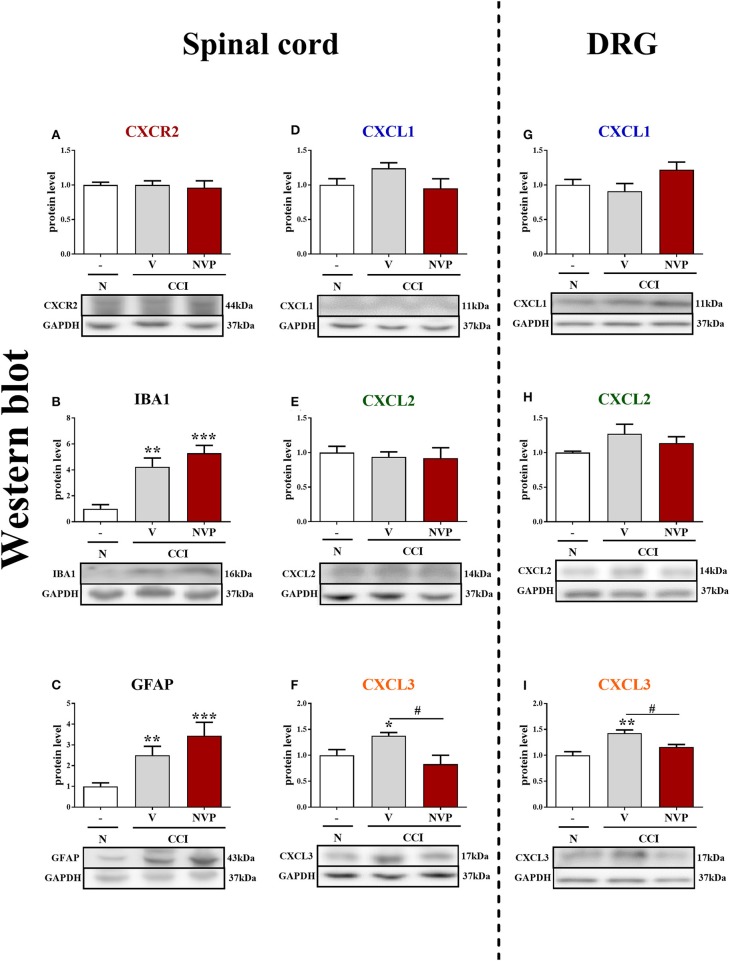
Effects of the repeated administration of NVP CXCR2 20 (NVP; 10 μg/5 μl; *i.t*.; 16 h and 1 h before CCI and then once a day for 7 days) on the protein levels of CXCR2, IBA1, GFAP, CXCL1, CXCL2, and CXCL3 proteins **(A–I)** in the spinal cord **(A–F)** and DRG **(G–I)** on the 7th day after CCI in rats. The data are presented as the mean fold changes relative to the control ± SEM (5–6 samples per group). Intergroup differences were analyzed using ANOVA with Bonferroni's multiple comparisons test. *p < 0.05, **p < 0.01, ***p < 0.001 indicate differences vs. naive rats. ^#^*p* < 0.05, indicate differences between V-treated and NVP-treated rats. CCI, chronic constriction injury; N, naive; V, vehicle; NVP, NVP CXCR2 20.

In the spinal cords of vehicle- and NVP-treated CCI-exposed rats, the level of the CXCR2 protein remained unchanged compared with that in the spinal cords of naive rats (Figure 5A). In the spinal cord of vehicle-treated, CCI-exposed rats, the levels of the IBA1 and GFAP proteins were increased (4.24-fold, *p* < 0.01; 2.5-fold, *p* < 0.01, respectively) compared with naïve rats (Figures 5B,C, respectively). NVP CXCR2 20 did not change the up-regulation of the IBA1 protein (5.3-fold in relation to control; Figure 5B) and GFAP protein (3.44-fold in relation to control; Figure 5C) levels in the spinal cord after CCI. No changes were observed in the spinal levels of the CXCL1 and CXCL2 proteins (Figures 5D,F, respectively), which corresponds well with the time-course study presented in Figures 1F,G. NVP CXCR2 20 also did not change the CXCL1 and CXCL2 protein levels (Figures 5D,F). Compared with naive rats, the level of the CXCL3 protein was increased 1.4-fold (*p* < 0.05) in vehicle-treated, CCI-exposed rats (Figure 5F), and importantly, NVP CXCR2 20 significantly attenuated CXCL3 protein expression to the level of control (1.7-fold; *p* < 0.05; Figure 5F).

In the DRG, as in the spinal cord, the levels of the CXCL1 and CXCL2 proteins remained unaltered in CCI-exposed rats, and NVP CXCR2 20 did not influence these factors (Figures 5G,H, respectively). The level of the CXCL3 protein was raised 1.4-fold (*p* < 0.01) in the vehicle-treated, CCI-exposed rats as compared to naive rats (Figure 5I), and again, the NVP CXCR2 20 significantly attenuated CXCL3 protein expression to the level of control (1.2-fold; *p* < 0.05) in the DRG (Figure 5I; Data Sheet 2).

### The Influence of the Single Intrathecal Administration of CXCL3-Neutralizing Antibody on Pain-Related Behaviors on the 7th Day After CCI in Mice

**Figure 6:**
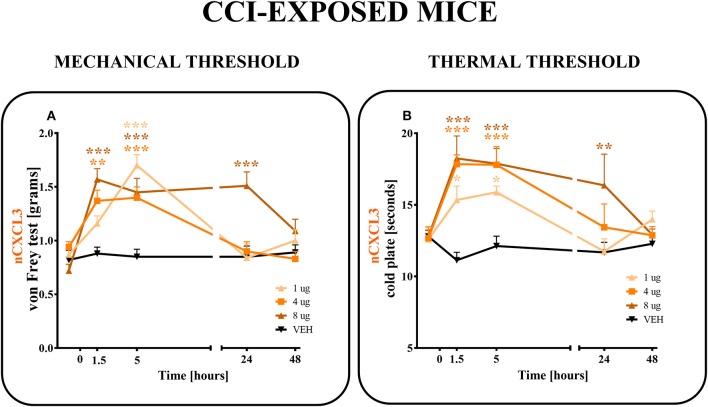
Effects of single administrations of CXCL3 neutralizing antibody **(A,B)** on nociceptive transmission in CCI-exposed mice. The effects of single intrathecal administrations of CXCL3 neutralizing antibody (1, 4, or 8 μg/5 μl) on mechanical hypersensitivity (von Frey test, **A**) and thermal hypersensitivity (cold plate test, **B**) were measured at 1.5, 5, 24, and 48 h after administration at 7 days after CCI. Data are presented as the means ± SEM (6–8 mice per group). The results were evaluated using one-way ANOVA followed by Bonferroni's test for comparisons of selected pairs. *p < 0.05, **p < 0.01, ***p < 0.001 for the comparison of CCI-exposed vehicle-treated animals with all groups at the indicated time points. Additionally, the results were evaluated using two-way ANOVA to determine the time × drug interaction (please see results in chapter 3.6). V, vehicle.

CXCL3-neutralizing antibodies were administered (*i.t*.) once on day 7 after CCI at the following concentrations: 1, 4 and 8 μg/5 μl (Figures 6A,B). The control group, CCI-exposed mice received vehicle (V; water for injection). Reactions to mechanical and thermal stimuli were assessed by von Frey (Figure 6A) and cold plate (Figure 6B) tests, respectively.

In the von Frey test, all doses (1, 4, and 8 μg) of CXCL3-neutralizing antibodies (Figure 6A) diminished the pain-related behavior. For the 1 μg dose (*p* < 0.001), the effect was observed only in the 5th hour (Figure 6A), while for doses 4 and 8 μg, the effect was already observed after 1.5 h (*p* < 0.01, *p* < 0.001; respectively) and strongly persisted after 5 h (*p* < 0.001). The analgesic effects of neutralizing antibody for doses 1 and 4 μg were reversed after 24 h and for the 8 μg dose only after 48 h, as measured by von Frey test (Figure 6A). Two-way ANOVA confirmed a significant interaction [*F*_(8, 103)_ = 5,555, *p* < 0.0001] between the investigated treatment for CXCL3-neutralizing antibody and investigated time points. The CXCL3-neutralizing antibody significantly increased the nociceptive threshold [*F*_(4, 103)_ = 12,65, *p* < 0.0001], showing an antinociceptive dose-dependent effect of the CXCL3-neutralizing antibody in the von Frey test.

In the cold plate test, after all doses (1, 4, and 8 μg) of CXCL3-neutralizing antibodies were used, the pain-related behavior was diminished (Figure 6B). This strong analgesic effect could be seen after 1.5 and 5 h for all tested doses (1, 4, and 8 μg) (*p* < 0.05; *p* < 0.001; *p* < 0.001, respectively) (Figure 6B). This effect was still observed after 24 h, but only for the highest dose, 8 μg (*p* < 0.01) (Figure 6B). The analgesic effects of 1 and 4 μg doses were reversed after 24 h as measured by the cold plate test (Figure 6B). Two-way ANOVA confirmed a significant interaction [*F*_(12, 111)_ = 2,723 *p* = 0.0029] between the investigated treatment for CXCL3-neutralizing antibody and the investigated time points. The CXCL3-neutralizing antibody significantly increased the nociceptive threshold [*F*_(4, 111)_ = 9,512 *p* < 0.0001], showing an antinociceptive dose-dependent effect of CXCL3-neutralizing antibody in the cold plate test.

The control antibody administration did not influence the development of tactile (e.g., pretest for V-treated group 0.67 ± 0.05 g vs. IgG-treated group 0.74 ± 0.07 g; 4 h after *i.t*. administration: V-treated group 0.83 ± 0.09 g vs. IgG-treated group 0.8 ± 0.08 g) or thermal (e.g., pretest for V-treated group 7.66 ± 0.5 s vs. IgG-treated group 7.71 ± 0.6 s; 4 h after *i.t*. administration: V-treated group 7.96 ± 0.5 s vs. IgG-treated group 8.4 ± 0.47 s) hypersensitivity.

### The Influence of Single Intrathecal Administration of CXCL3 Preceded by NVP CXCR2 20 Injection on Nociceptive Transmission in Naive Mice

**Figure 7:**
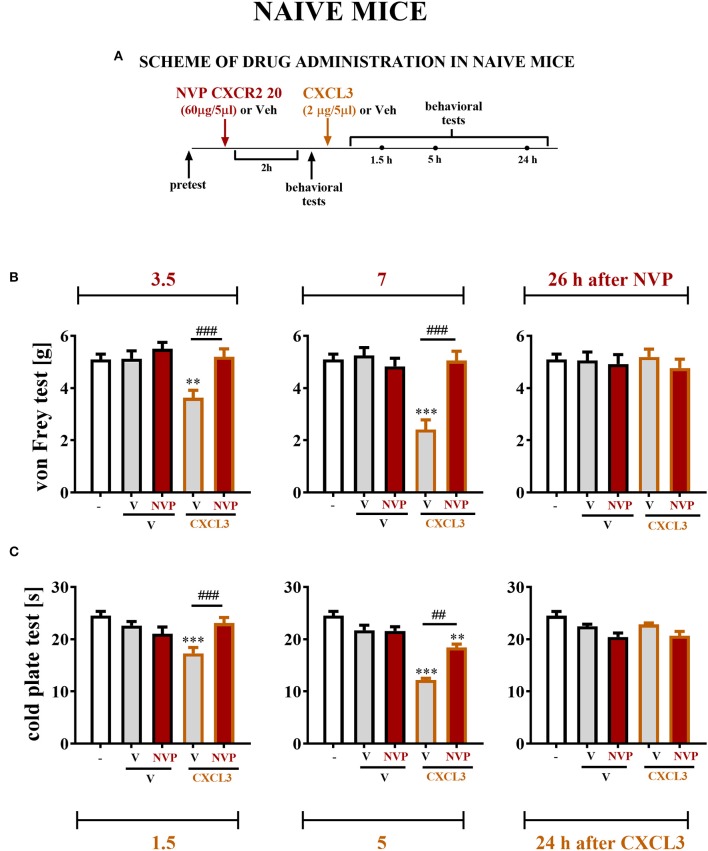
Effects of single NVP CXCR2 20 administration on a single CXCL3 injection and nociceptive transmission in naive mice **(B,C)**. Single intrathecal administrations of vehicle (V) or NVP CXCR2 20 (60 μg/5 μl) were performed 120 min before a single intrathecal administration of V or CXCL3 (2 ng/5 μl). The effects of administrations on mechanical (von Frey test; **B**) and thermal (cold plate test; **C**) hypersensitivity were measured 3.5, 7 and 26 h after the NVP CXCR2 20 injection (1.5, 5, and 24 h after the CXCL3) **(A)**. Data are presented as the means ± SEM (6–8 mice per group). The results were evaluated using one-way ANOVA followed by Bonferroni's test for comparisons of selected pairs. **p < 0.01, ***p < 0.001 indicate differences in comparison with V+V-treated animals at the indicated time points. ^*##*^*p* < 0.01, ^*###*^*p* < 0.001 indicate differences in comparison with V+CXCL3-treated animals at the indicated time points. V, vehicle; NVP, NVP CXCR2 20.

The reactions to non-noxious (Figure 7B) and noxious (Figure 7C) stimuli in naive, vehicle + vehicle–treated and vehicle + NVP–treated (60 μg/5 μl) mice were similar (Figures 7B,C). At 2 h after substance administration, behavioral tests were conducted, and the mice received CXCL3 (2 ng/5 μl) following the testing (Figure 7A). The behavioral tests were performed at 1.5, 5, and 24 h after CXCL3 injection (3.5, 7, and 26 h after NVP CXCR2 20 administration) (Figure 7A). The vehicle + CXCL3–treated group (800 ng/5 μl) developed mechanical and thermal hypersensitivity (Figures 7B,C, respectively), which was prevented by pretreatment with NVP CXCR2 20 (Figures 7B,C).

### The Influence of NVP CXCR2 20 on the Levels of the CXCL1, CXCL2, and CXCL3 Proteins in Rat Microglial and Astroglial Cell Cultures at 24 h After Lipopolysaccharide Stimulation

**Figure 8:**
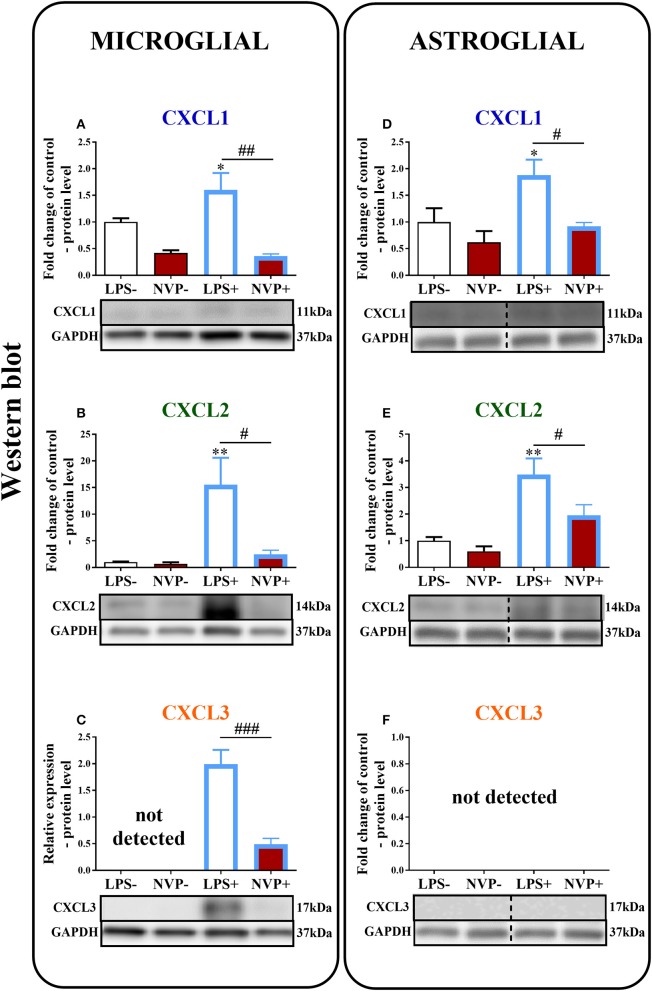
Effects of on NVP CXCR2 20 levels of the CXCL1, CXCL2, and CXCL3 proteins **(A–F)** in primary rat microglial **(A–C)** and astroglial **(D–F)** cell cultures. Samples were analyzed 24 h after cells were stimulated with LPS. The data are presented as the fold change relative to the control and relative protein levels. *Fold change relative to control*: the Western blot data are presented as the means ± SEM and represent the normalized averages derived from analyses of 3–4 independent experiments. Intergroup differences were analyzed using ANOVA with Bonferroni's multiple comparisons test. *p < 0.05, **p < 0.01, ***p < 0.001 indicate differences in comparison with the control group (vehicle-treated non-stimulated cells); ^#^*p* < 0.05, ^*##*^*p* < 0.01, ^*###*^*p* < 0.001 indicate differences between vehicle-treated and NVP-treated LPS-stimulated cells. *Relative protein level:* Inter-group differences in relative protein level were analyzed using a t-test. ^*###*^*p* < 0.001 indicates differences compared to the vehicle-treated LPS-stimulated cells. LPS-, vehicle-treated non-stimulated cells; NVP-, NVP-treated non-stimulated cells, LPS+, vehicle-treated LPS-stimulated cells; NVP+, NVP-treated LPS-stimulated cells. In **(D–F)** the blots are cropped which was shown with a dotted line on the representation bands below the figures.

In microglial cell cultures, we observed an expressive increase in the levels of CXCL1 (1.6-fold, *p* < 0.05; Figure 8A) and CXCL2 (15.6-fold, *p* < 0.01; Figure 8B) proteins at 24 h after LPS stimulation. CXCL3 protein levels were not detected in non-stimulated cells, but we observed strongly increased CXCL3 protein levels in LPS-stimulated microglia (Figure 8C). NVP CXCR2 20 decreased the CXCL1 (4.4-fold, *p* < 0.01; Figure 8A), CXCL2 (6.3-fold, *p* < 0.05; Figure 8B), and CXCL3 (4.3-fold, *p* < 0.001; Figure 8C) protein levels in LPS-stimulated cells compared with those in vehicle-treated LPS-stimulated microglia.

In astroglial cell cultures, we observed a considerable increase in the levels of the CXCL1 (1.9-fold, *p* < 0.05; Figure 8D) and CXCL2 (3.5-fold, *p* < 0.01; Figure 8E) proteins at 24 h after LPS stimulation. NVP CXCR2 20 decreased the CXCL1 (2-fold, *p* < 0.05; Figure 8D) and CXCL2 (1.8-fold, *p* < 0.05; Figure 8E) protein levels in LPS-stimulated cells compared with those in vehicle-treated LPS-stimulated microglia. CXCL3 protein levels were not detected in astrocytes (in non-stimulated or LPS-treated) (Figure 8F; Data Sheet 3).

### The Influence of Single Administrations of NVP CXCR2 20 on Opioid Effectiveness on the 7th Day Post-CCI in Rats

**Figure 9:**
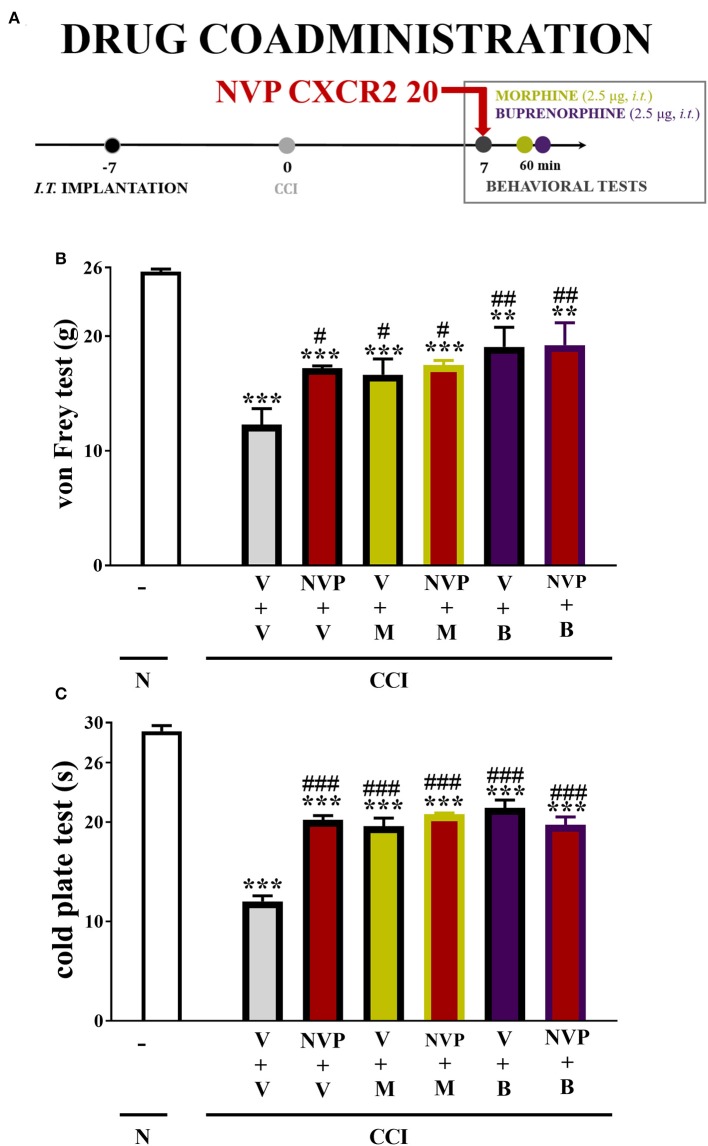
Scheme of drug co-administration **(A)**. Effects of single **(B,C)** administration of NVP CXCR2 20 (NVP; 10 μg/5 μl; single dose *i.t*.; on the 7th day post-CCI) **(A)** on pain-related behaviors (von Frey test **A**; cold plate test **B**) and the analgesic effects of morphine (M; 2.5 μg/5 μl; single dose *i.t*.; on the 7th day post-CCI, 4 h after NVP or V injection) and buprenorphine (B; 2.5 μg/5 μl; single dose *i.t*.; on the 7th day post-CCI, 4 h after NVP or V injection) on CCI-exposed rats. The data are presented as the means ± SEM of 6 rats per group. Intergroup differences were analyzed using ANOVA with Bonferroni's multiple comparisons test. **p < 0.01, ***p < 0.001 indicate differences compared with naïve rats. ^#^*p* < 0.05, ^*##*^*p* < 0.01, ^*###*^*p* < 0.001 indicate differences compared V+V-treated, CCI-exposed rats. B, buprenorphine; CCI, chronic constriction injury; M, morphine; N, naive; NVP, NVP CXCR2 20; V, vehicle.

In the von Frey test, single injections of the respective opioids caused similar analgesic effects as single injections of NVP CXCR2 20 (10 μg/5 μl). The combined administration of NVP CXCR2 20 and morphine (2.5 μg/5 μl) or buprenorphine (2.5 μg/5 μl) did not change the effectiveness of the individual substances (Figure 9B).

In the cold plate test, single injections of the respective opioids caused similar analgesic effects as single injections of NVP CXCR2 20 (10 μg/5 μl). The single administration of the combination of NVP CXCR2 20 and morphine (2.5 μg/5 μl) or buprenorphine (2.5 μg/5 μl) did not change their efficacy (Figure 9C).

## Discussion

First, we observed that intrathecal injections of CINCs induced pain-related behaviors in naive mice, which is related to the CXCR2 neuronal response. Second, RT-qPCR and Western blot results of the time course changes in chemokines indicated CXCL3 involvement in the development of neuropathic pain, whereas only the mRNA expression of the two other ligands was increased in the initial phase. Moreover, the neutralizing antibody for CXCL3 reduced neuropathic pain symptoms in mice on day 7 after CCI. Third, immunofluorescence staining indicated that in the spinal cord, CXCR2 and CXCL3 are expressed mainly in neurons as measured at 7 days after sciatic nerve injury. Fourth, we proved that a potent and selective CXCR2 receptor antagonist, NVP CXCR2 20, reduces the symptoms of neuropathic pain and the CCI-upregulated levels of CXCL3 in the spinal cord and DRG and prevents the development of hypersensitivity to stimuli after CXCL3 administration. Finally, we provided evidence that the chronic intrathecal administration of NVP CXCR2 20 did not attenuate microglial activation, and this is probably the reason why these compounds do not enhance morphine/buprenorphine analgesia, which was observed in our previous studies on the CXCR3 antagonist (±)-NBI-74330 (29). Notably, to the best of our knowledge, this study is the first to present the comparison of these three chemokines in a single experiment involving a neuropathic pain model. Our findings provide evidence that, out of all investigated CINCs, spinal CXCL3 plays an important role in CXCR2 signaling in neuropathic pain.

Our results obtained in a neuropathic pain model are consistent with other findings (58–61), which suggests that CXCR2 is important for nociception transmission. First, it was shown that the expression of CXCR2 becomes upregulated in macrophages and neutrophils infiltrated locally at a nerve injury site (62). Immunohistochemical studies demonstrated that under neuropathic conditions, the majority of spinal CXCR2 molecules are located on dorsal horn neurons (15, 63); however, their upregulation also occurs in non-neuronal cells (15, 63, 64). Second, it has recently been published that under homeostatic conditions, spinal microglia do not express CXCR2, but it can be upregulated upon its activation in central nervous system (CNS) pathologies, such as Alzheimer's disease, multiple sclerosis, traumatic brain or nerve injuries, and inflammation, including Complete Freund's Adjuvant injection (15, 65–70). Our immunohistochemical staining proved the presence of spinal CXCR2 in neurons. Our results show the upregulation of CXCR2 mRNA on days 2, 7, and 14 after CCI in rats, which is in line with Xu et al. (26). The protein changes in CXCR2 are not measurable, which is not surprising because many GPCRs may rapidly internalize upon agonist stimulation and subsequently become replaced by newly synthesized receptors. Like other GPCRs, CXCR2 is rapidly internalization following a burst of agonist-mediated signaling. The mechanism appears to be similar to that used by many other GPCRs (71). During receptor activation, the induction of internalization of CXCR2 depends on the interactions between the N-terminal of the chemokine and the N-domain of the chemokine receptor (72). After agonist removal, internalized CXCR2 is associated with different cellular trafficking regulators and may be recycled to the cell surface, thereby enabling a subsequent round of signaling (71, 73) or may enter lysosomal sorting pathway of CXCR2 (74). These various receptor-mediated events are directly dependent on the CXCL1 or CXCL2 concentration (75), and dysregulation of these processes e.g., in the pathogenesis of neuropathic pain could switch the cell phenotype. Changes in ligand levels during neuropathic pain may lead to disorders in receptor activation and signaling, but this requires further study. In our recent studies using the CCR4 antagonist (Kujacz et al., submitted), we observed an increase in mRNA levels, in parallel with no change in protein levels in the neuropathic pain model. However, blocking these receptors causes strong analgesic effects—this requires further molecular studies—while the importance of these receptors in the nociceptive transmission is beyond doubt, as is CXCR2. The neuronal location of CXCR2 suggested by some authors (15, 63, 70, 76) correlates well with our behavioral results and explains why intrathecally injected CXCR2 ligands induce very fast and strong pain-like behavior in naive mice. Kiguchi et al. (62) reported that the administration of the histone acetyltransferase inhibitor anacardic acid suppressed the upregulation of CXCR2 at an injured sciatic nerve site after its partial ligation. Liang et al. (77) showed that the spinal administration of the CXCR2/CXCR1 antagonist, SCH527123, potently reversed sensitization after traumatic brain injury. Our study provides, for the first time, evidence that intrathecal injections of a potent and selective CXCR2 antagonist, NVP CXCR2 20, reduce neuropathic pain symptoms by modulating the release of CXCL3 at the spinal cord and DRG level. Based on these results and previously published data, we propose that the spinal blockade of CXCR2 signaling may produce efficient analgesic effects under neuropathy.

CXCL1 was the first discovered endogenous ligand of CXCR2. However, its role in nociception remains unclear. Our results indicate that in naive mice, the intrathecal administration of a high dose (800 ng) of CXCL1 causes hypersensitivity to mechanical and/or thermal stimuli, which suggests a confirmed spinal neuronal location of CXCR2. In 2007, Li et al. (78) reported that injuries of the spinal cord and sciatic nerve induce the upregulation of CXCL1 in DRG neurons (76) at 3 but not 7 days after surgery. Subsequently, it was shown that CXCL1 sensitizes primary neurons by triggering an increase in calcium ion influx, modulating potassium and sodium currents (15, 59, 79–81). In the CNS, however, CXCL1 astrocyte expression has already been shown in animal models after brain (82) and spinal cord (15, 83) injury, as well as in humans with multiple sclerosis (84). This observation was later confirmed by *in vitro* results showing that CXCL1 is released from primary astroglial cells following TNFα (15, 85) and IL-1beta (84) stimulation. Similarly, we showed the LPS-induced release of CXCL1 from primary astroglia cultures and, for the first time, microglial cells. Nevertheless, under neuropathic pain conditions, CXCL1 mRNA does not increase in parallel with microglia activation (10, 25, 44). We observed a spinal increase in CXCL1 mRNA only on day 2 after sciatic nerve injury, similar to Manjavachi et al. (86) after partial sciatic nerve ligation in Swiss mice. In our experiments, the spinal protein changes of CXCL1 in Wistar rats remained undetectable, which corresponds to the results in BALB/c mice after spinal nerve transection (87). Nevertheless, after spinal nerve ligation, some authors observed elevated CXCL1 protein levels in Sprague-Dawley rats and Albino Swiss mice (15, 88). Such discrepancies may arise due to the applied model of neuropathic pain or as a result of the specific genomic characteristics of the abovementioned rodent strains.

CXCL1 is 90% identical in amino acid sequence to its related chemokine, CXCL2. Based on our data, we were the first to indicate that, similar to other CINCs, the intrathecal administration of CXCL2 causes the rapid development of hypersensitivity to thermal and mechanical stimuli in naive mice, which confirms the spinal neuronal location of CXCR2. In 2012, Haraguchi et al. (89) showed that CXCL2 is produced in an injured sciatic nerve by partial ligation and suggested that this chemokine is secreted by monocytes and acts as chemotactic for leukocytes, which was confirmed by Kiguchi et al. (62). These authors observed that CXCL2 mRNA became elevated in an injured sciatic nerve during the first 24 h after damage, but no further changes were detected until day 14. Similarly, we showed an increase in CXCL2 mRNA at the spinal cord level only shortly (day 2) after sciatic nerve injury. However, the spinal protein level of CXCL2 did not change after injury, suggesting that under neuropathy, CXCL2 plays an important role in the PNS (peripheral nervous system) rather than the CNS. The lack of spinal CXCL2 upregulation was unexpected, since earlier *in vitro* studies showed an increase in CXCL2 in activated mouse primary microglia (89), which is in agreement with our *in vitro* results obtained in rat microglia and astroglia cultures. Nonetheless, we did not observe any spinal upregulation of CXCL2 protein in parallel with glial activation under neuropathic pain, as measured on days 2–28. Based on the literature and our current data, we hypothesize that the induction of the CXCL2/CXCR2 axis is extremely important at the periphery after nerve injury but probably not at the spinal cord level.

CXCL3 is another member of CINCs, and it is the least researched chemokine in the context of nociception processes. Despite the fact that structural details and receptor binding interactions in the case of CXCL1 and CXCL2 have been elucidated for years, the information regarding the structural and biophysical characteristics of CXCL3 became available as late as 2018, when Gulati et al. (30) successfully cloned, expressed, and purified the recombinant CXCL3. The authors revealed that although the overall structural and oligomerization features of CXCL3 and CXCL1/2 are similar, prominent differences can be observed on their characteristic surface structures, thus indicating a functional divergence. CXCL3/CXCR2 signaling exerts its functions through a number of signaling pathways, including p38MAPK, ERK1/2, and JAK2/STAT3 (90, 91). The involvement of these pathways in the development of neuropathy has been known for many years, also in our model (11, 39, 40). CXCL3 is strongly expressed in a number of tumorous conditions (30, 92); however, its role in the context of neuropathy has yet to be studied. Our results regarding time course changes of mRNA and protein indicate that of all CINCs, CXCL3 is the most important in the development of neuropathic pain, and its protein level undergoes upregulation up to 7 days. In addition, our results regarding naive mice showed for the first time that intrathecal CXCL3 administrations cause hypersensitivity to mechanical and/or thermal stimuli, appearing quickly after injection and lasting up to 24 h because of its location in neurons, what can be confirmed by immunofluorescent staining. What is more, the antibody neutralization of endogenous CXCL3 results in reductions of these symptoms in mice on day 7 after CCI. Moreover, we showed that CXCL3-induced pain behavior is abolished by pretreatment with NVP CXCR2 20, which proves an important role for CXCR2 in the effects of this chemokine. CXCL1 and CXCL2 are strongly related to each other, both structurally and functionally. They play a pivotal role in the immune response by recruiting and activating neutrophils in PNS with the highest concentration 1–3 days after injury (93, 94). CXCL3 helps neutrophil recruitment to inflamed areas and functions as an important mediator of macrophage chemotaxis (95). Our results provide the first evidence that spinal CXCL3 plays an important role in the development of neuropathic pain, since its protein is the only protein whose upregulation can be observed 2–7 days after CCI. Within the same time frame, we were able to observe the strongest microglia activation (10, 44). Additionally, our *in vitro* studies were the first to indicate the release of CXCL3 by stimulated microglial primary cells. In the case of CINCs, it is not expressed in the microglia at rest, and LPS strongly induces its release, suggesting that CXCL3 may act as a proinflammatory factor in activated microglia by insults (e.g., infection, injury, stress). Our immunohistochemical staining indicates mainly the neuronal origin of CXCL3 in the spinal cord, however, what's interesting, we observe the release of CXCL3 by activated microglial cells on day 7 after injury. The *in vitro* and *in vivo* results suggest that the microglia cells are able to produce this compound in some circumstances, which requires further in-depth research. On the other hand, CXCL3, as a strongly pronociceptive mediator, is not produced by astrocytes, which play an important role in restoring homeostasis in the CNS (96, 97). Our results indicate for the first time an important contribution of CXCL3 both in the initiation and development of neuropathic pain, and modulation of CXCL3 release can have beneficial effects, which may help in relieving the symptoms of neuropathic pain.

While opioids are commonly used in the treatment of chronic pain, in neuropathic pain they exhibit rather weak effectiveness (98). Previous reports suggest that, the CXCR2 receptor is capable of forming heterodimers with opioid receptors, and a change in the conformation of receptors may have an effect on their activation or ligand binding (99). The interaction of CXCR2 with DOR has been confirmed so far (99), which, however, may not be sufficient and significant for the effectiveness of morphine and buprenorphine during neuropathic pain. In the literature, it is well-established that microglial activation is essential for opioid analgesia under neuropathic pain (8, 100–103). It has been shown that the activation of microglia and subsequent increased level of pronociceptive cytokines, which have anti-opioid properties, e.g., IL-1beta (104), IL-18 (10) decreased opioid effectiveness and the development of morphine tolerance (8, 105). Recently, we have shown that blockade of CCR2 [RS504393, (25)], CCR5 [maraviroc, (28)], and CXCR3 [(±)-NBI-74330]; (29) can restore the analgesic activities of morphine and/or buprenorphine. Therefore, initially, it was surprising that the CXCR2 antagonist NVP CXCR2 20 did not enhance the analgesia of these opioids. However, in contrast to the antagonists of CCR2, CCR5, and CXCR3 (25, 28, 29), repeated administration of NVP CXCR2 20 did not diminish spinal microglia activation as well as important kinases associated with the activation of these cells, e.g., p38MAPK, ERK1/2 (own unpublished results). Our earlier findings support the view that activated spinal microglia through the modulation of the production of cytokines, including chemokines, are important not only in the development of neuropathic pain but also in a diverse efficacy of opioid analgesics (106–109).

## Conclusions

As far as we are concerned, our study is the first to show strong pronociceptive properties of CXCL3. Moreover, chronic administrations of the CXCR2 antagonist (NVP CXCR2 20) can diminish hypersensitivity (and simultaneously CXCL3 expression) at the spinal cord and DRG level in a rat neuropathic pain model. Importantly, NVP CXCR2 20 does not influence microglia or astroglia activation, and probably for this reason, this substance is not responsible for increasing opioid analgesia under neuropathic pain. In summary, neuronal spinal CXCL3-CXCR2 signaling plays a crucial role in the pathogenesis of neuropathy after peripheral nerve injury, and we propose this site of action as a promising target for enabling the inhibition of its development in patients suffering from neuropathic pain. However, more research is needed on the role of all CXCR2 ligands (including CXCL8), not just those of the CINC family.

## Data Availability

The datasets generated for this study are available on request to the corresponding author.

## Ethics Statement

The number of animals was limited to the necessary minimum. The experiments were carried out in compliance with IASP recommendations (35), NIH Guide for Care and Use of Laboratory Animals and approved by the 2nd Local Ethical Committee on Animal Testing in Maj Institute of Pharmacology, Polish Academy of Sciences (12 Smetna Str., 31-343 Krakow, Poland; permission number: 1277/2015 and 262/2017).

## Author Contributions

All authors have made substantial contributions to the conception, design of the study, analysis and interpretation of data for the present study, final approval of the version to be published, and agreement to be accountable for all aspects of the research in ensuring that questions related to the accuracy or integrity of any part of the study is appropriately investigated and resolved. AP, ER, KP, GK, AC, WM, and JM made the experiments. AP and JM planned the study. AP, ER, KP, GK, AC, WM, IN, and JM analyzed and interpreted the results, drafted the manuscript, and accepted the finalized version.

### Conflict of Interest Statement

The authors declare that the research was conducted in the absence of any commercial or financial relationships that could be construed as a potential conflict of interest.
