# Supplementary figures and images for: Pharmacological Blockade of Spinal CXCL3/CXCR2 Signaling by NVP CXCR2 20, a Selective CXCR2 Antagonist, Reduces Neuropathic Pain Following Peripheral Nerve Injury
Source: Front Immunol. 2019 Sep 26;10:2198. doi: 10.3389/fimmu.2019.02198 (PMC6775284; doi:10.3389/fimmu.2019.02198)

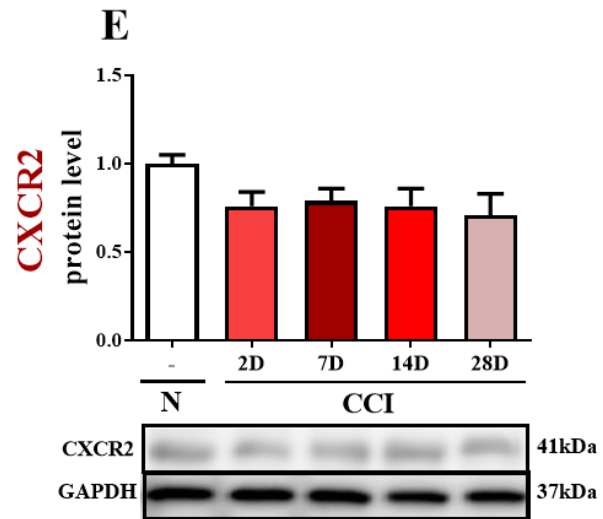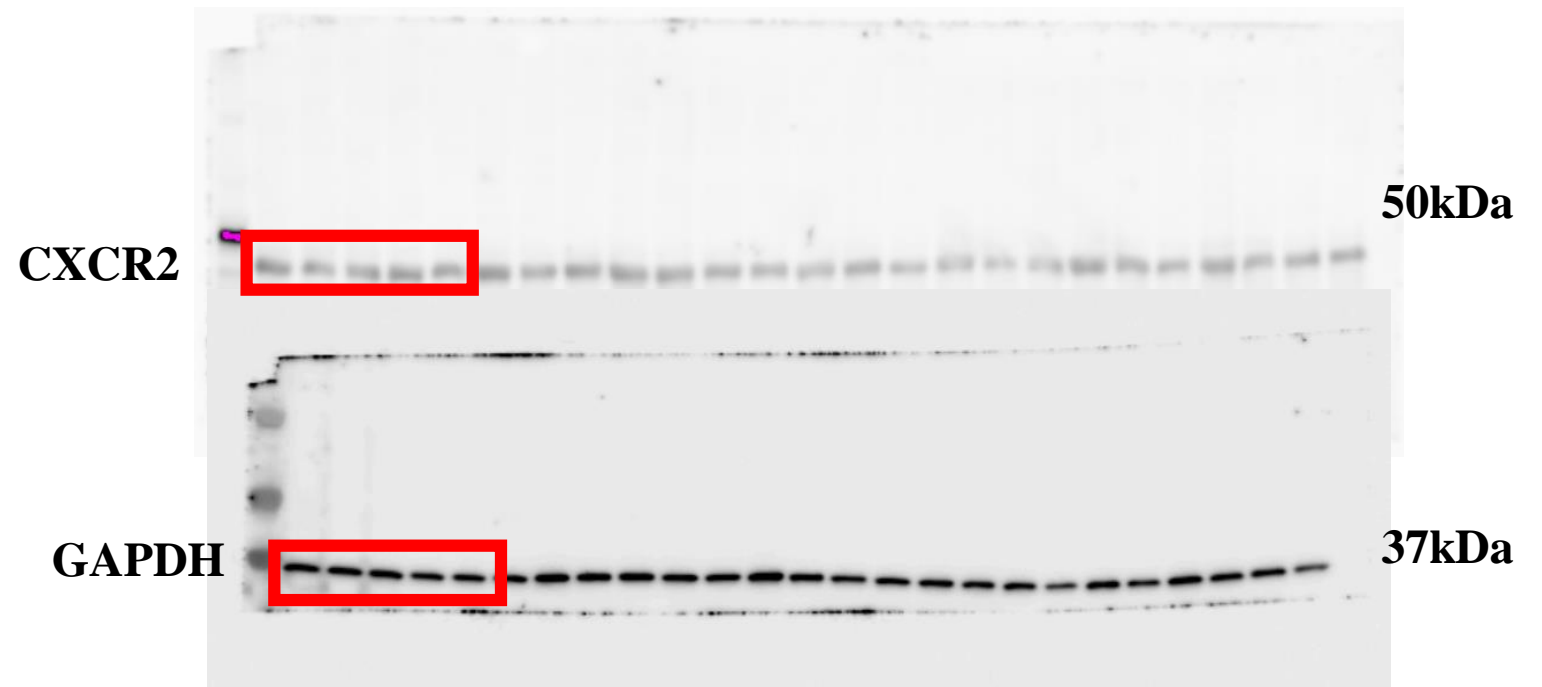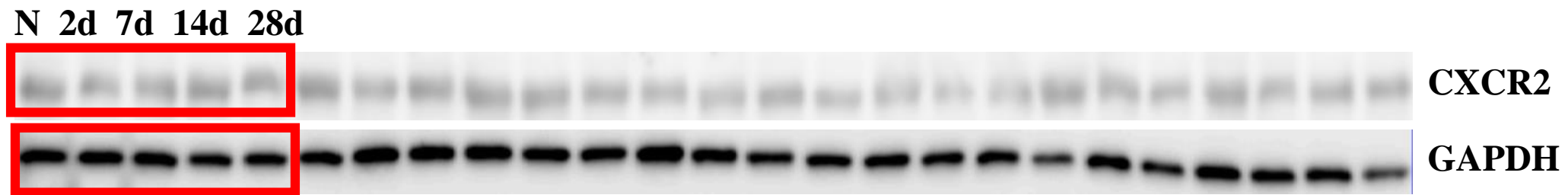

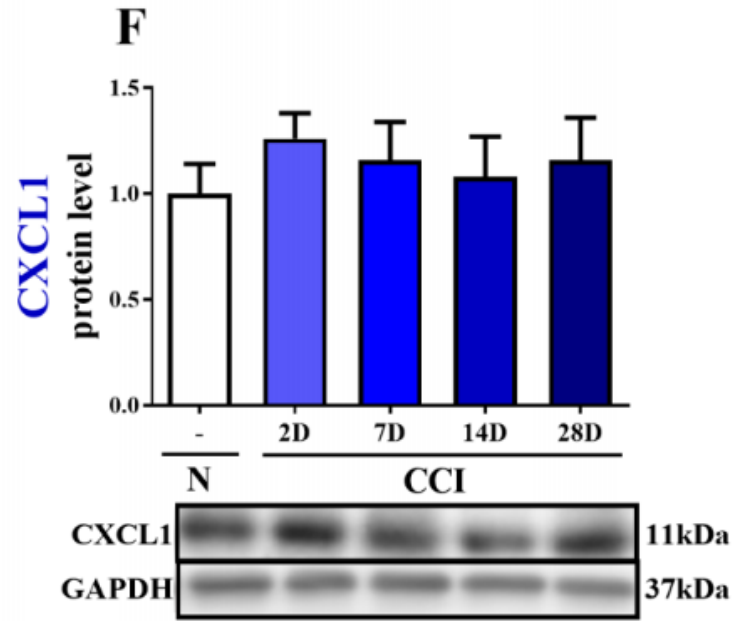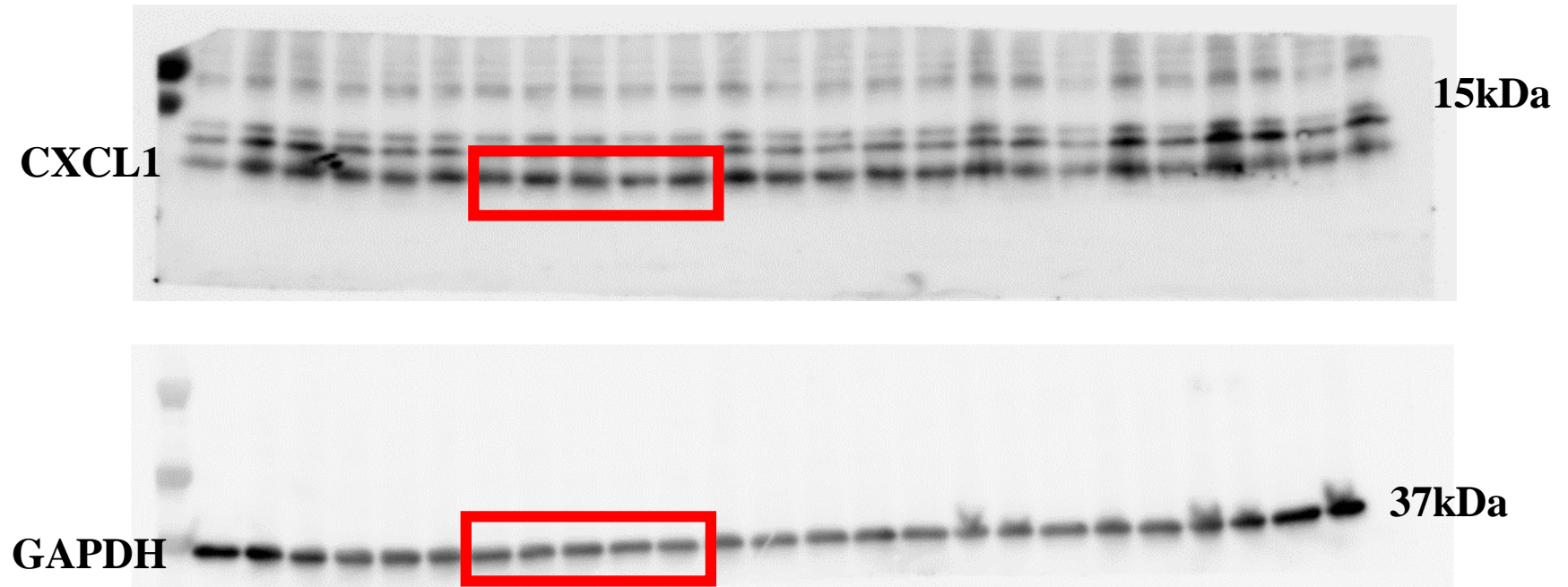

N 2d 7d 14d 28d

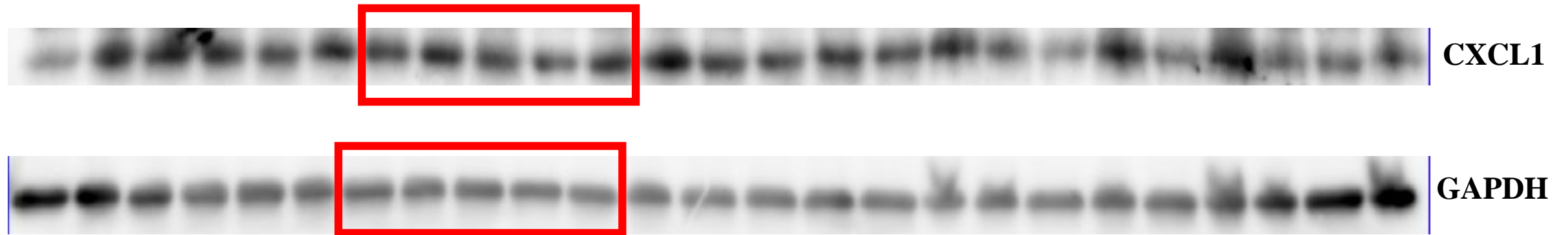

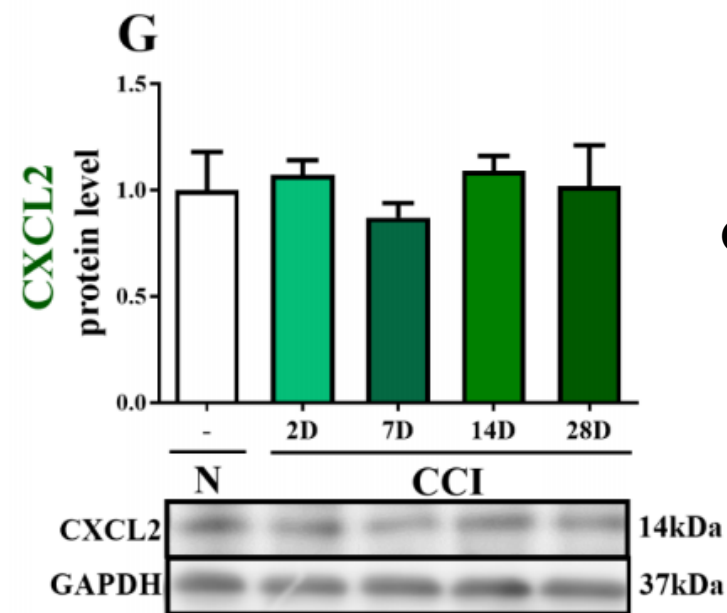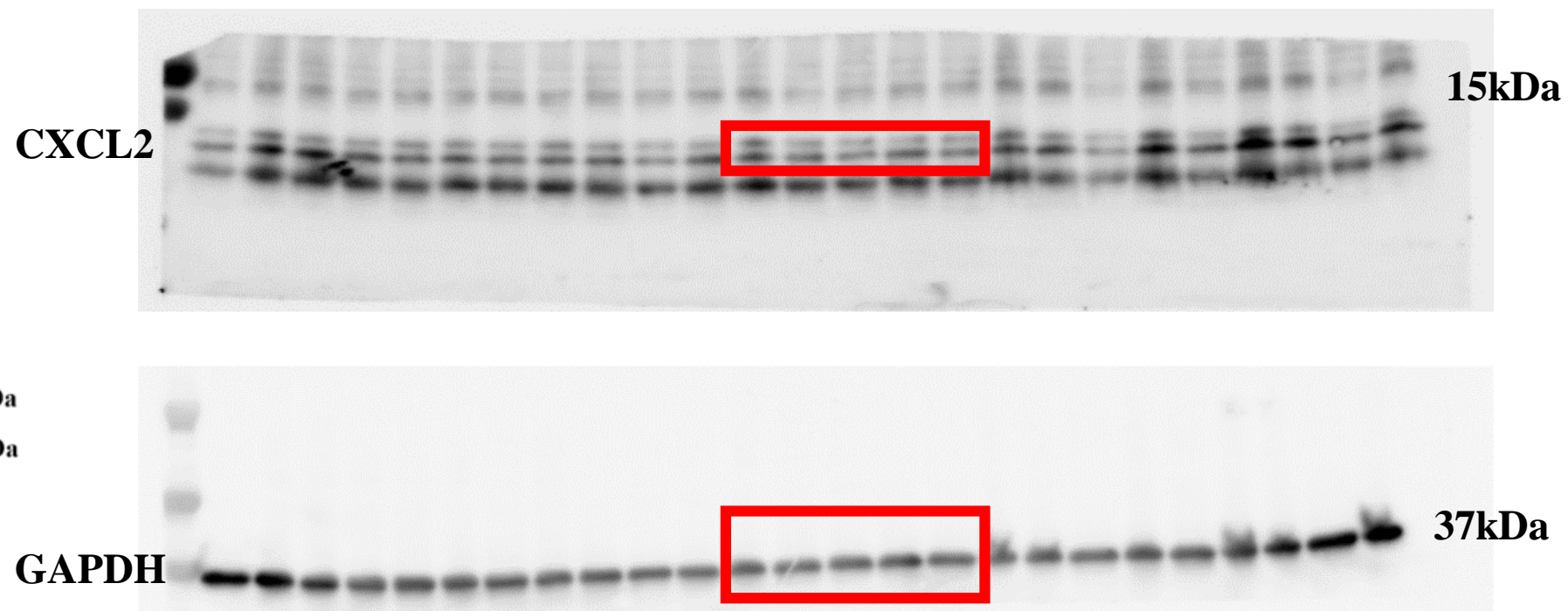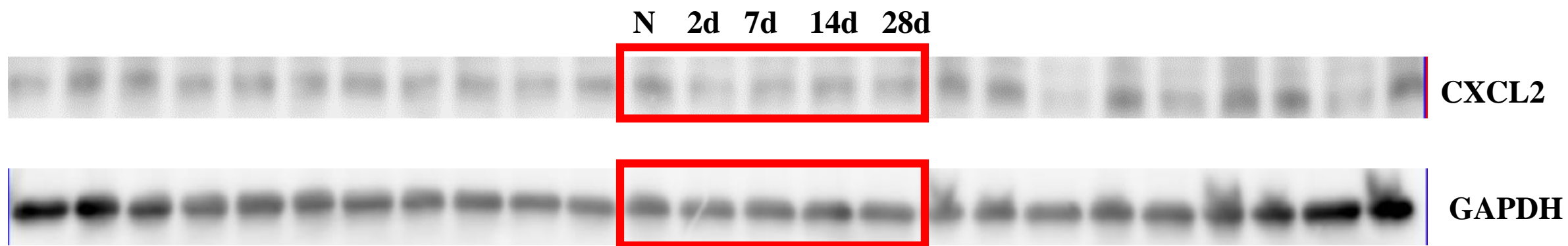

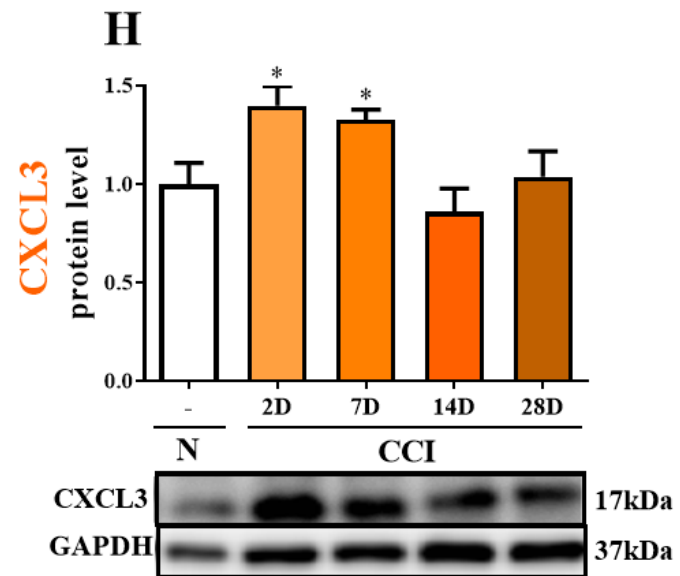

GAPDH

20kDa

CXCL3

37kDa

N 2d 7d 14d 28d

CXCL3

GAPDH

Supplement: Supplementary file 1 [file Data_Sheet_1.PDF]
