# Supplementary material for: Pharmacological Blockade of Spinal CXCL3/CXCR2 Signaling by NVP CXCR2 20, a Selective CXCR2 Antagonist, Reduces Neuropathic Pain Following Peripheral Nerve Injury
Source: Front Immunol. 2019 Sep 26;10:2198. doi: 10.3389/fimmu.2019.02198 (PMC6775284; doi:10.3389/fimmu.2019.02198)
Supplement: Supplementary file 2 [file Data_Sheet_2.PDF]

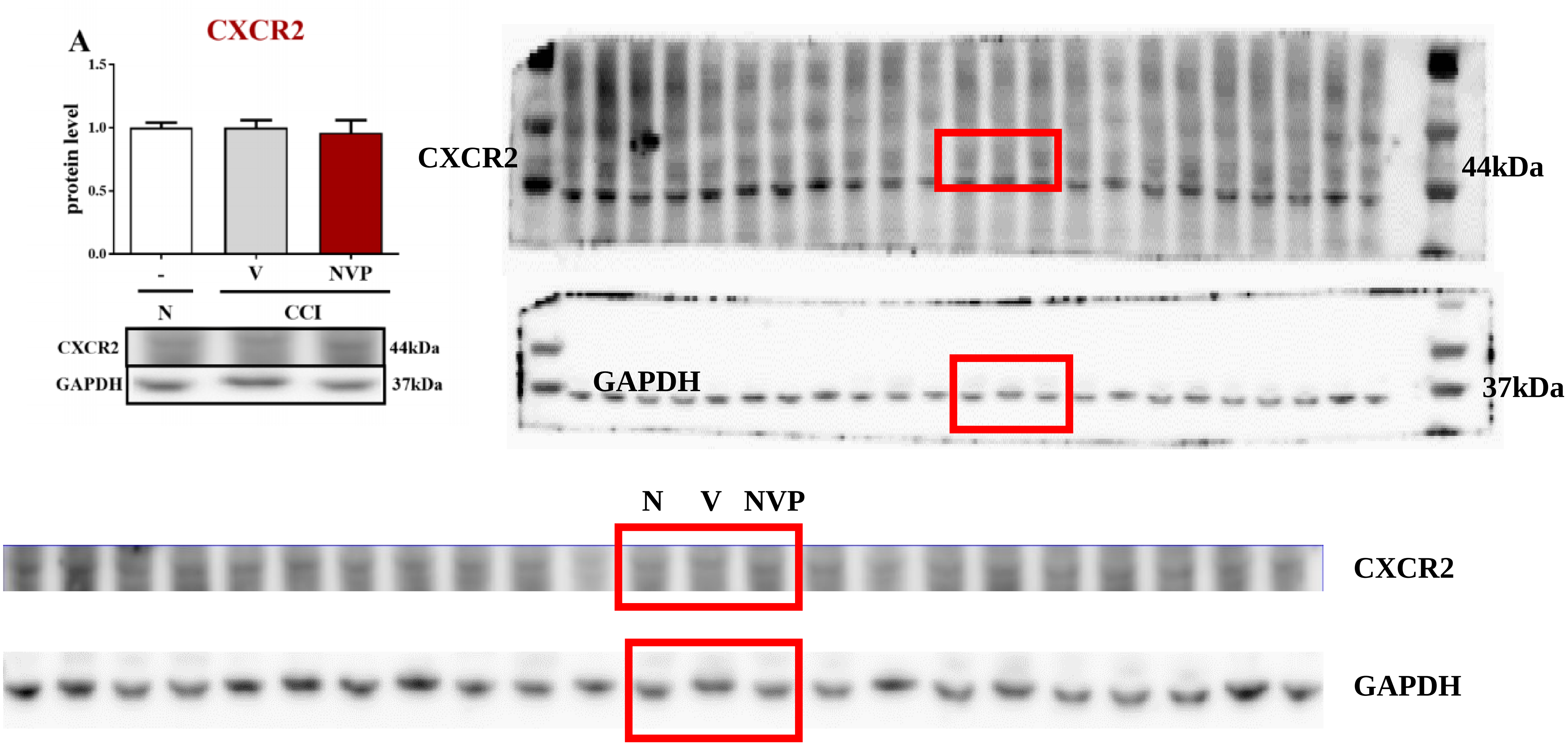
A
CXCR2
protein level
1.5
1.0
0.5
0.0
-
V
NVP
N
CCI
CXCR2
GAPDH
44kDa
37kDa
CXCR2
44kDa
GAPDH
37kDa
N
V
NVP
CXCR2
GAPDH

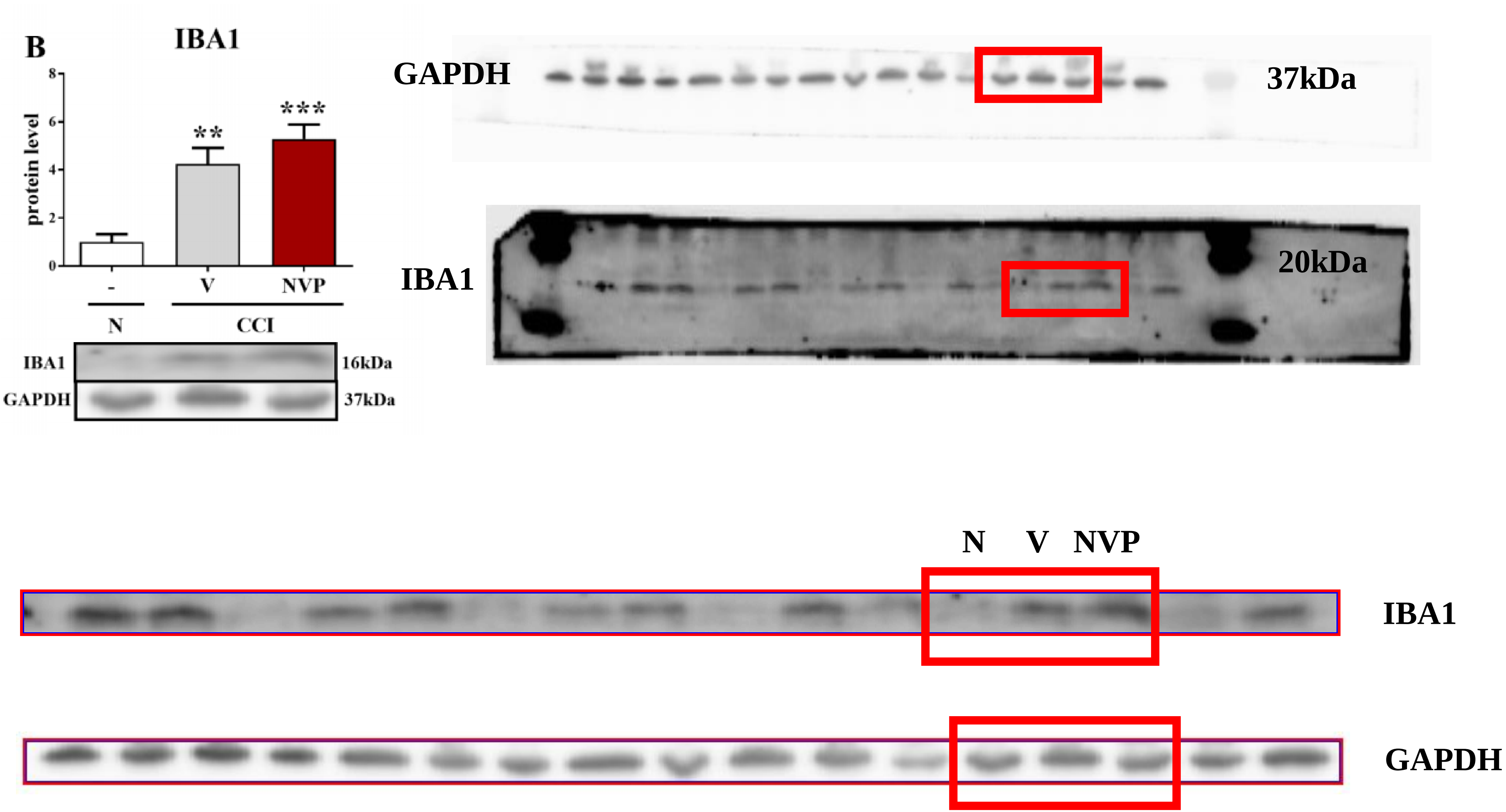
B
IBA1
protein level
8
6
4
2
0
**
***
-
V
NVP
N
CCI
IBA1
16kDa
GAPDH
37kDa
GAPDH
37kDa
IBA1
20kDa
N
V
NVP
IBA1
GAPDH

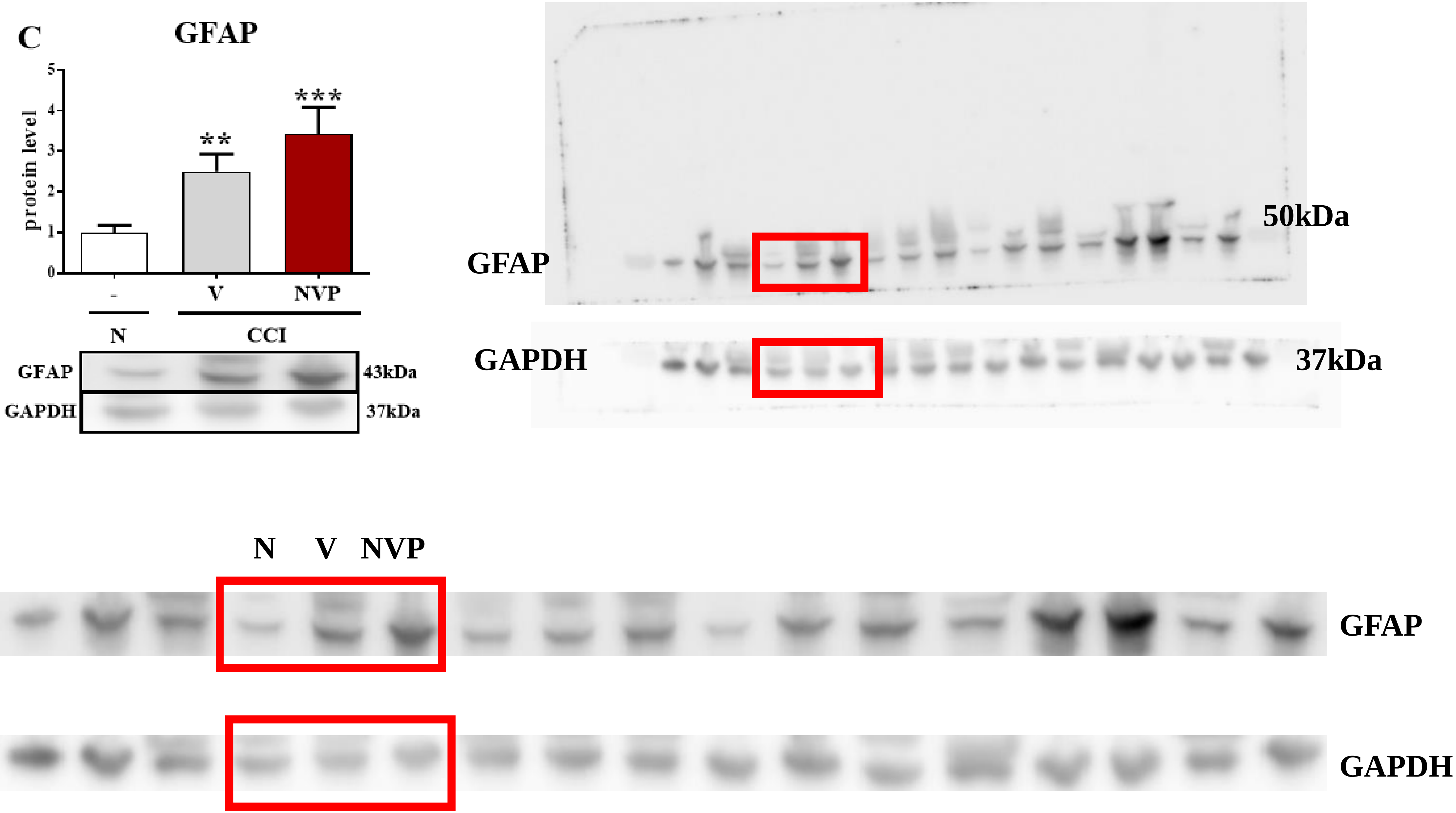

C
GFAP
protein level
0
1
2
3
4
5
**
***
-
V
NVP
N
CCI
GFAP
GAPDH
43kDa
37kDa
GFAP
GAPDH
50kDa
37kDa
N
V
NVP
GFAP
GAPDH

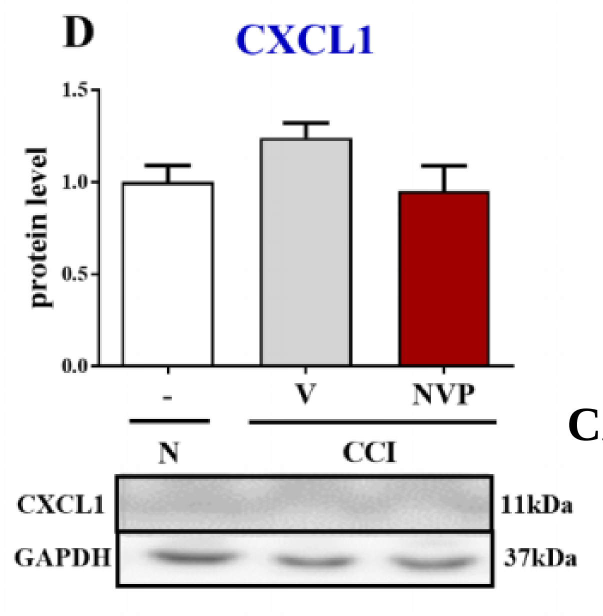
D
CXCL1
1.5
1.0
0.5
0.0
protein level
-
V
NVP
N
CCI
CXCL1
11kDa
GAPDH
37kDa

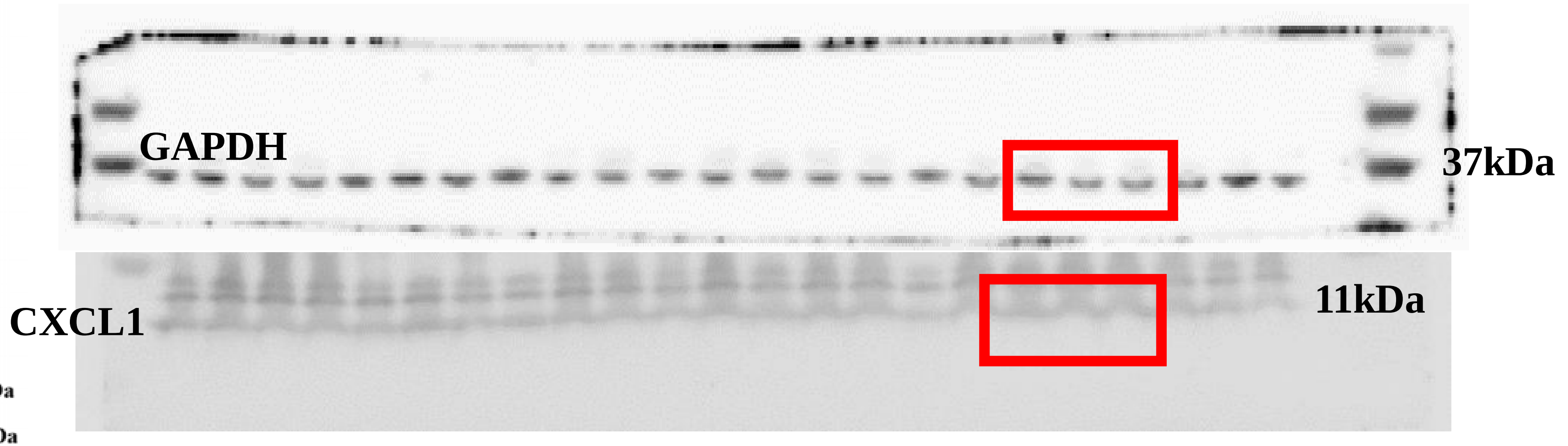
GAPDH
37kDa
CXCL1
11kDa

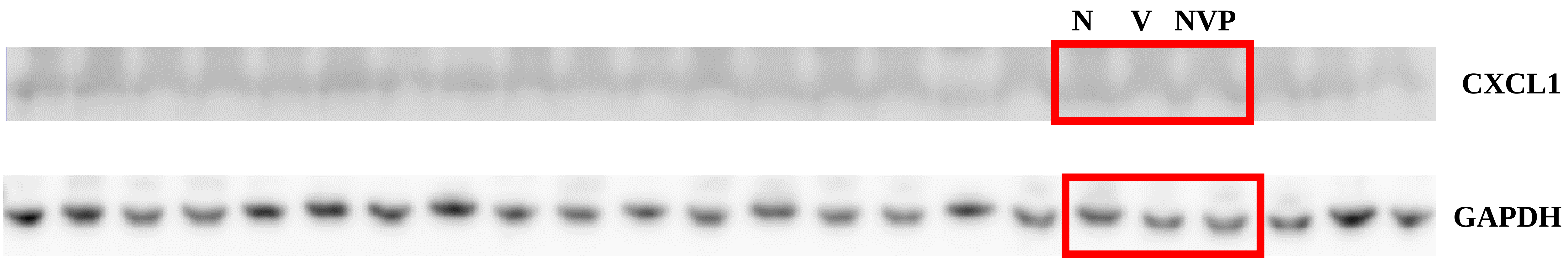
N
V
NVP
CXCL1
GAPDH

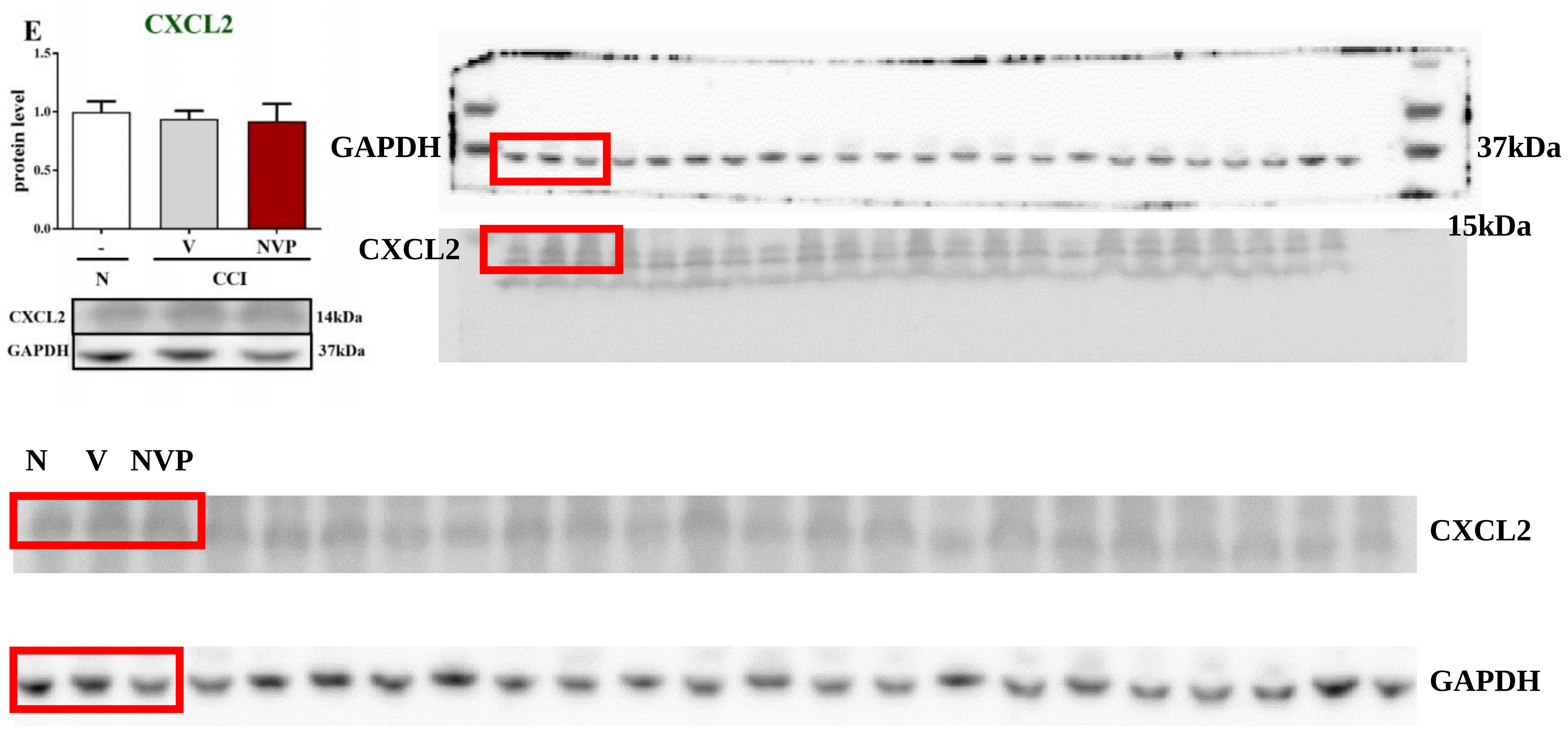
E
CXCL2
protein level
1.5
1.0
0.5
0.0
-
V
NVP
N
CCI
CXCL2
14kDa
GAPDH
37kDa
GAPDH
37kDa
15kDa
CXCL2
N
V
NVP
CXCL2
GAPDH

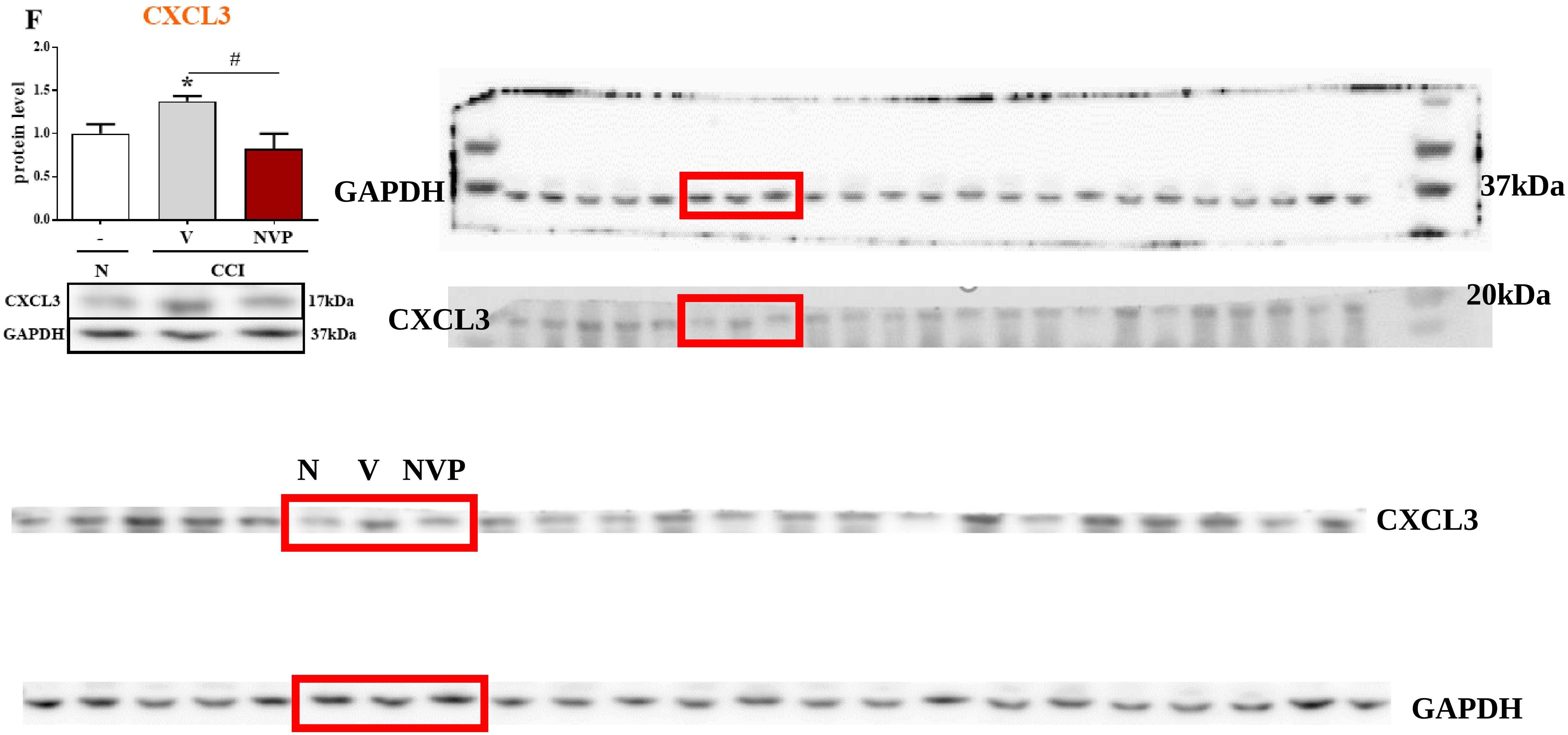
F
CXCL3
protein level
2.0
1.5
1.0
0.5
0.0
#
*
-
V
NVP
N
CCI
CXCL3
17kDa
GAPDH
37kDa
GAPDH
37kDa
CXCL3
20kDa
N V NVP
CXCL3
GAPDH

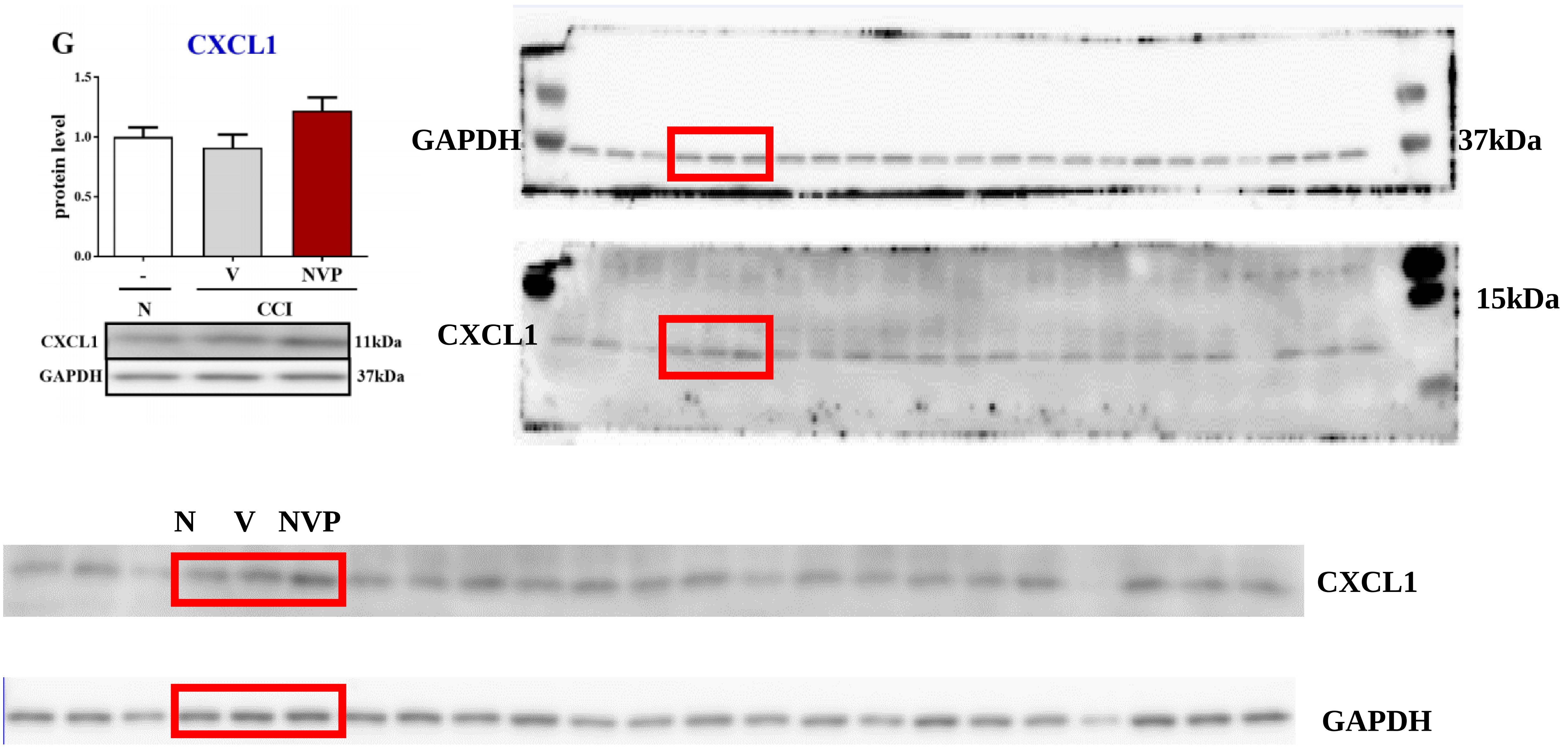
G
CXCL1
protein level
1.5
1.0
0.5
0.0
-
V
NVP
N
CCI
CXCL1
11kDa
GAPDH
37kDa
GAPDH
37kDa
CXCL1
15kDa
N
V
NVP
CXCL1
GAPDH

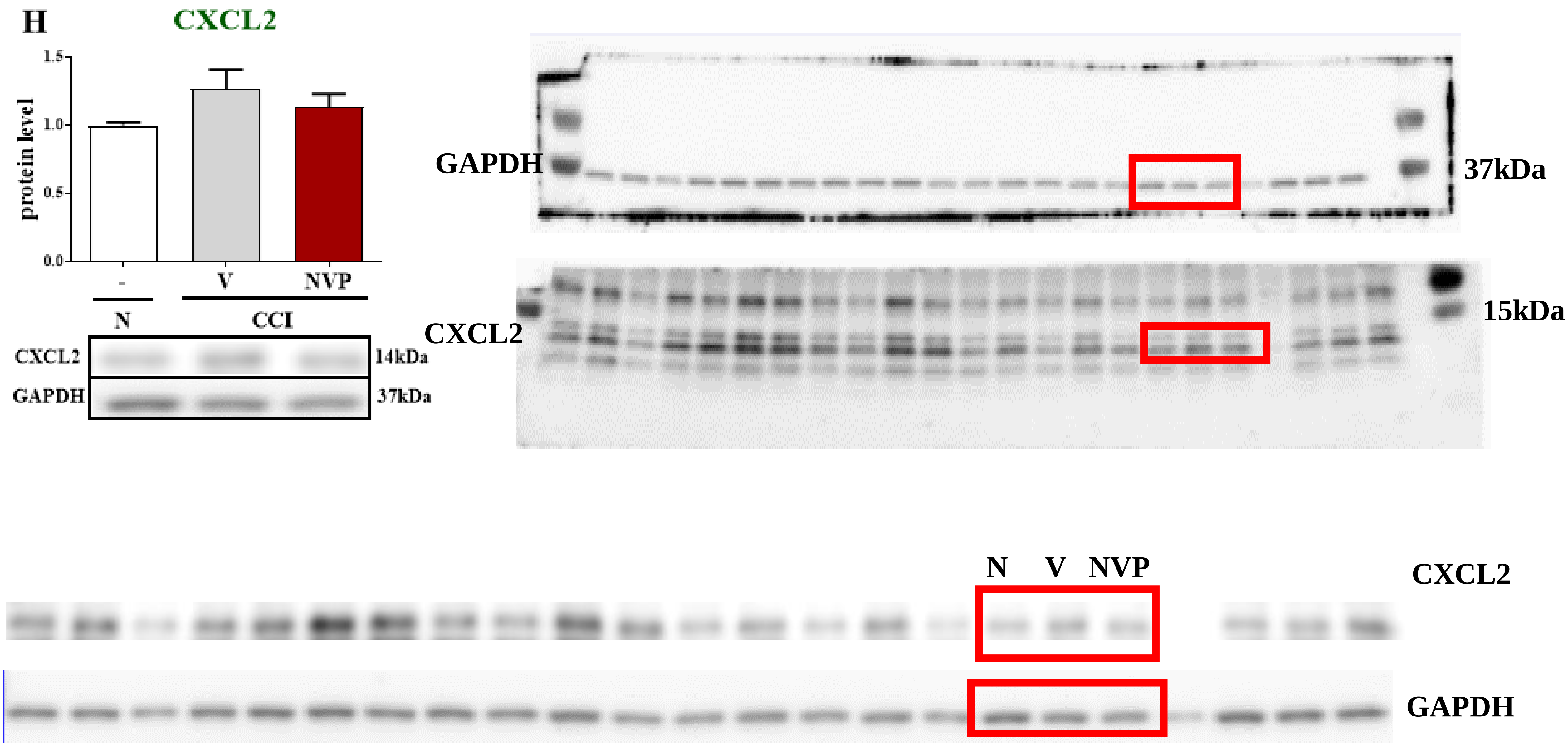
H
CXCL2
protein level
1.5
1.0
0.5
0.0
-
V
NVP
N
CCI
CXCL2
14kDa
GAPDH
37kDa
GAPDH
37kDa
CXCL2
15kDa
N
V
NVP
CXCL2
GAPDH

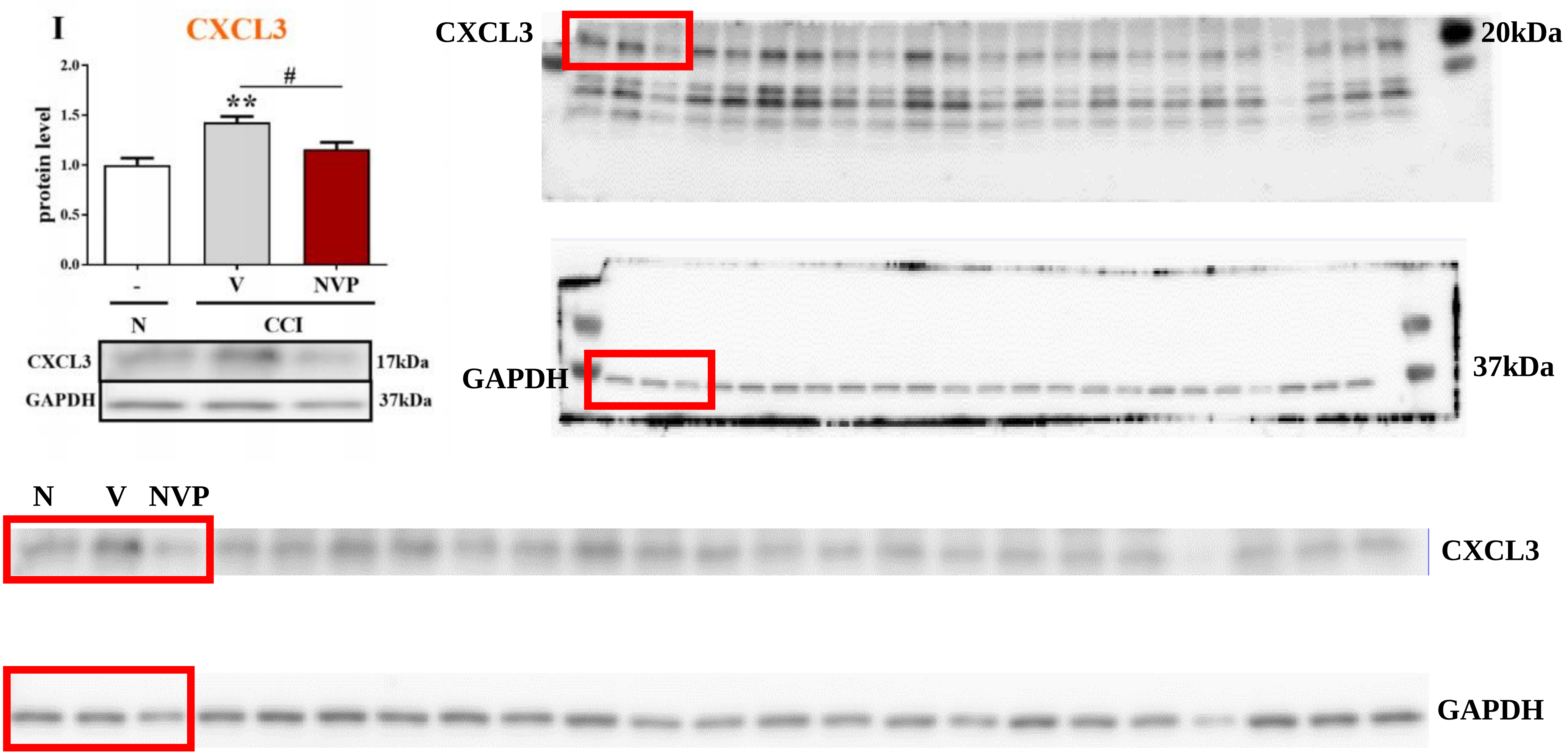
I
CXCL3
protein level
2.0
1.5
1.0
0.5
0.0
**
#
-
V
NVP
N
CCI
CXCL3
GAPDH
17kDa
37kDa
CXCL3
20kDa
GAPDH
37kDa
N
V
NVP
CXCL3
GAPDH
