# Supplementary material for: Pharmacological Blockade of Spinal CXCL3/CXCR2 Signaling by NVP CXCR2 20, a Selective CXCR2 Antagonist, Reduces Neuropathic Pain Following Peripheral Nerve Injury
Source: Front Immunol. 2019 Sep 26;10:2198. doi: 10.3389/fimmu.2019.02198 (PMC6775284; doi:10.3389/fimmu.2019.02198)
Supplement: Supplementary file 3 [file Data_Sheet_3.PDF]

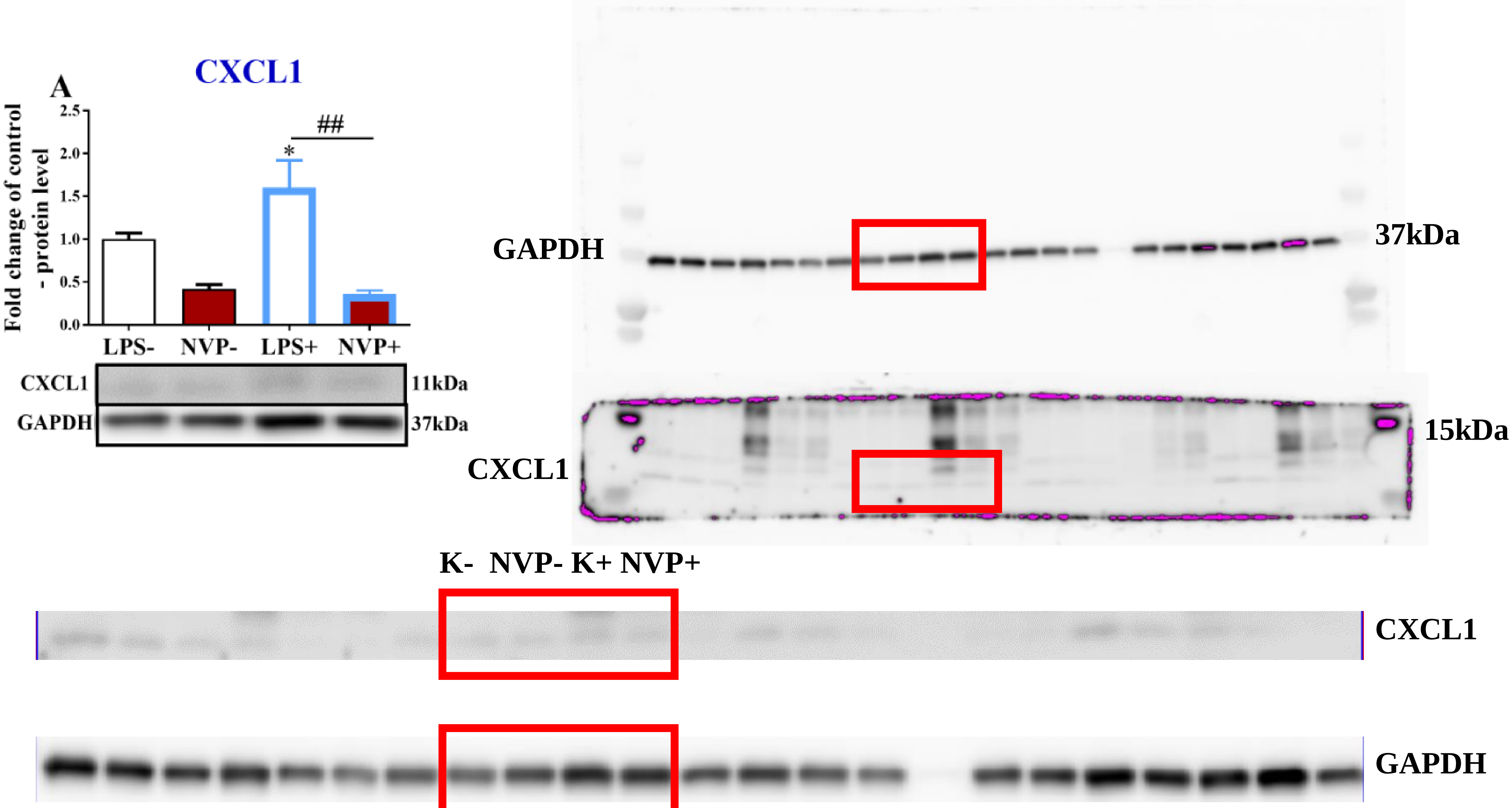

key symbols:

K-/LPS- – vehicle-treated non-stimulated cells ; NVP- - NVP-treated non-stimulated cells

K+/LPS+ – LPS-stimulated cells; NVP+ - NVP-treated LPS-stimulated cells

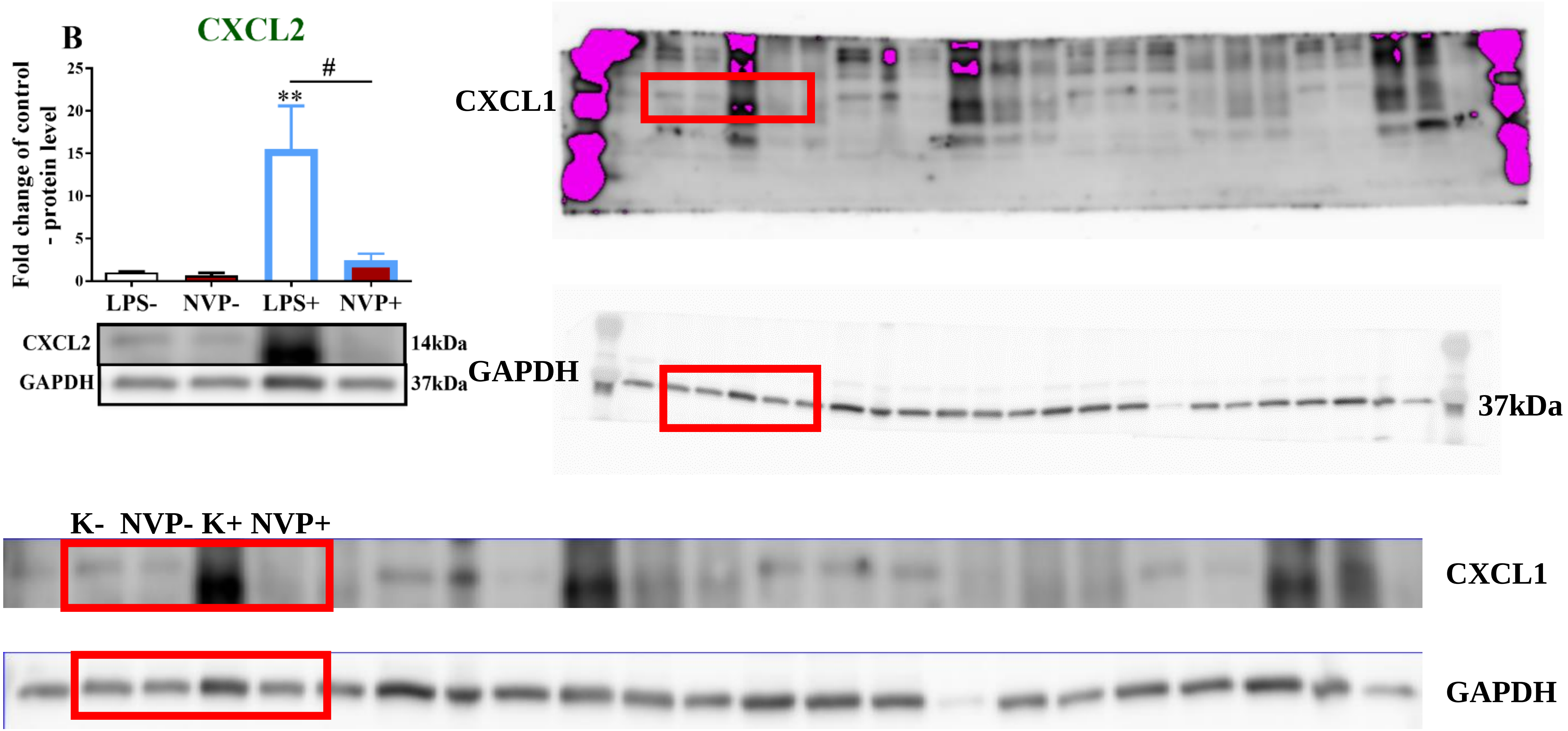

key symbols:

K-/LPS- – vehicle-treated non-stimulated cells ; NVP- - NVP-treated non-stimulated cells

K+/LPS+ – LPS-stimulated cells; NVP+ - NVP-treated LPS-stimulated cells

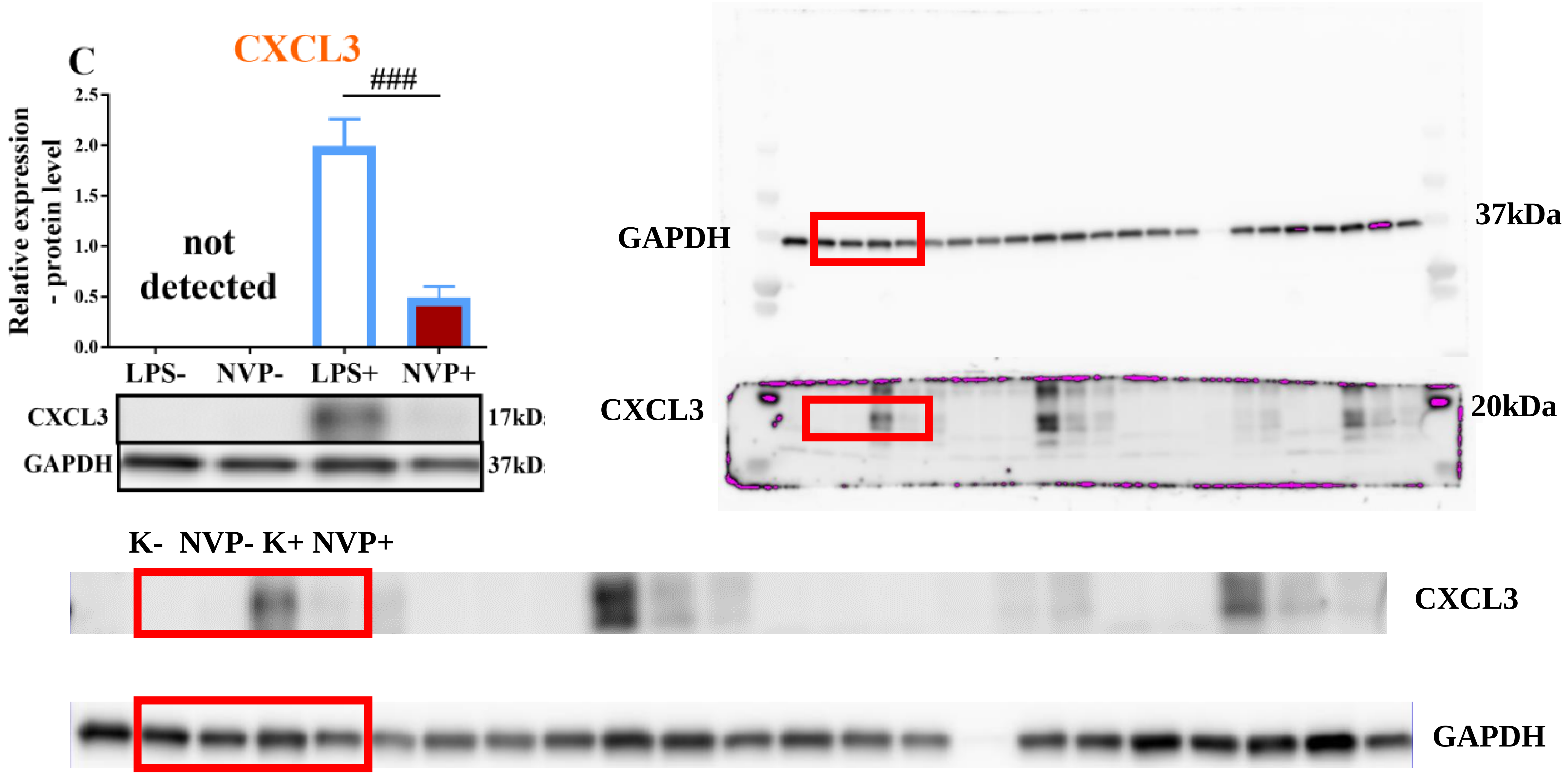

key symbols:

K-/LPS- – vehicle-treated non-stimulated cells ; NVP- - NVP-treated non-stimulated cells

K+/LPS+ – LPS-stimulated cells; NVP+ - NVP-treated LPS-stimulated cells

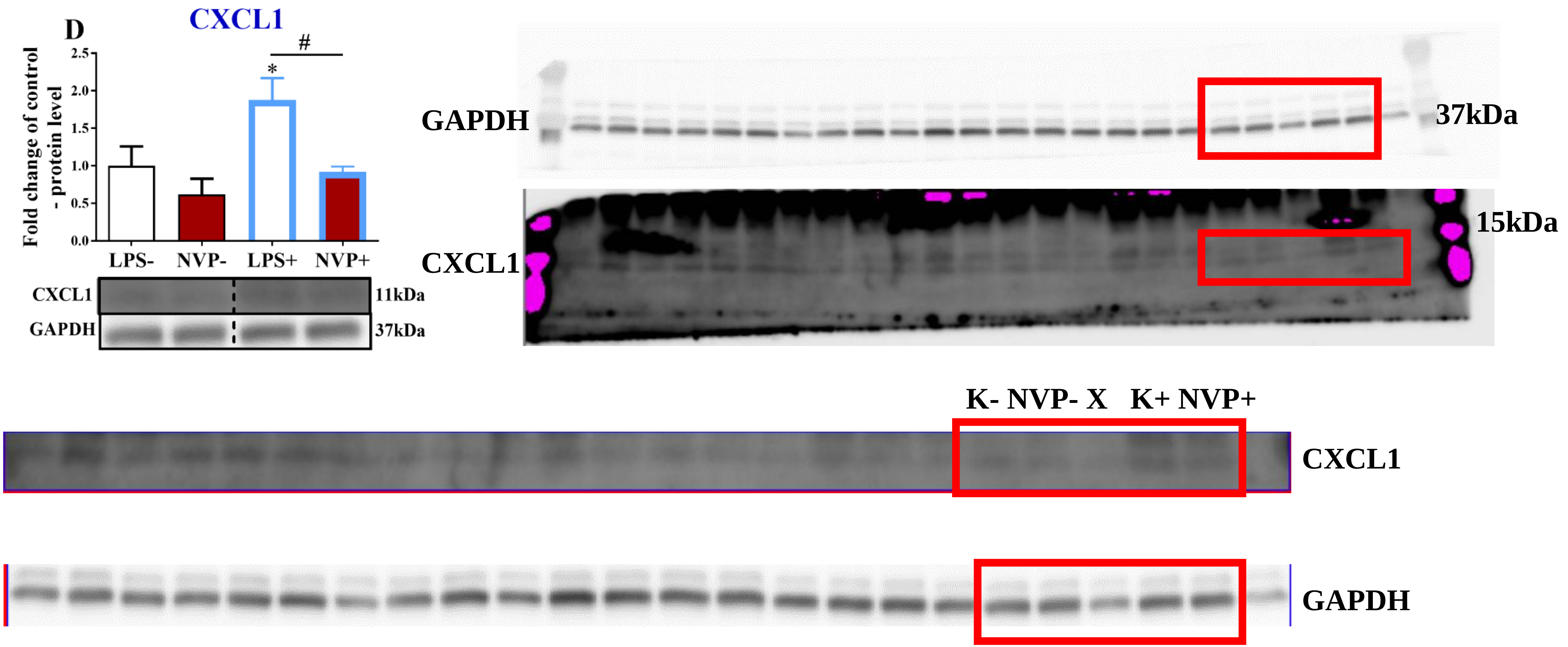

key symbols:

K-/LPS- – vehicle-treated non-stimulated cells ; NVP- - NVP-treated non-stimulated cells

K+/LPS+ – LPS-stimulated cells; NVP+ - NVP-treated LPS-stimulated cells

X - another compound

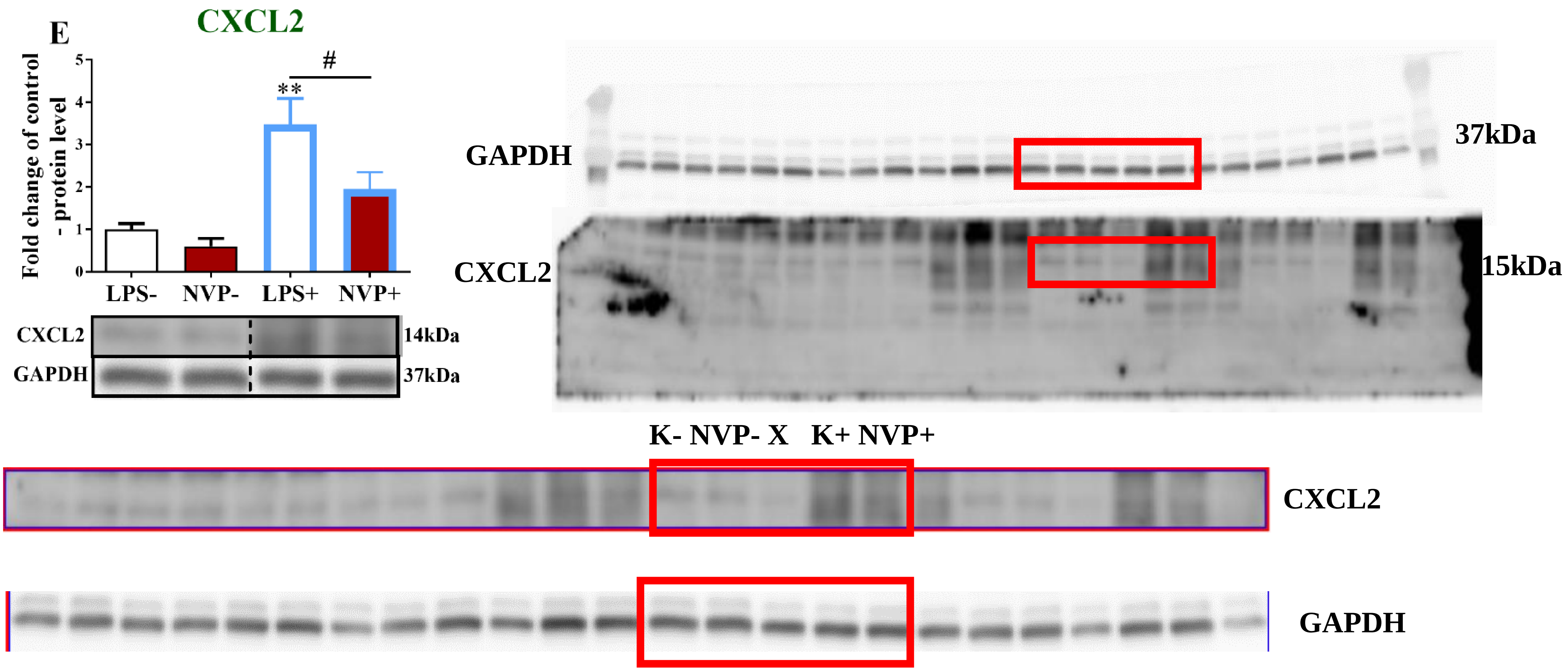

key symbols:

K-/LPS- – vehicle-treated non-stimulated cells ; NVP- - NVP-treated non-stimulated cells

K+/LPS+ – LPS-stimulated cells; NVP+ - NVP-treated LPS-stimulated cells

X - another compound

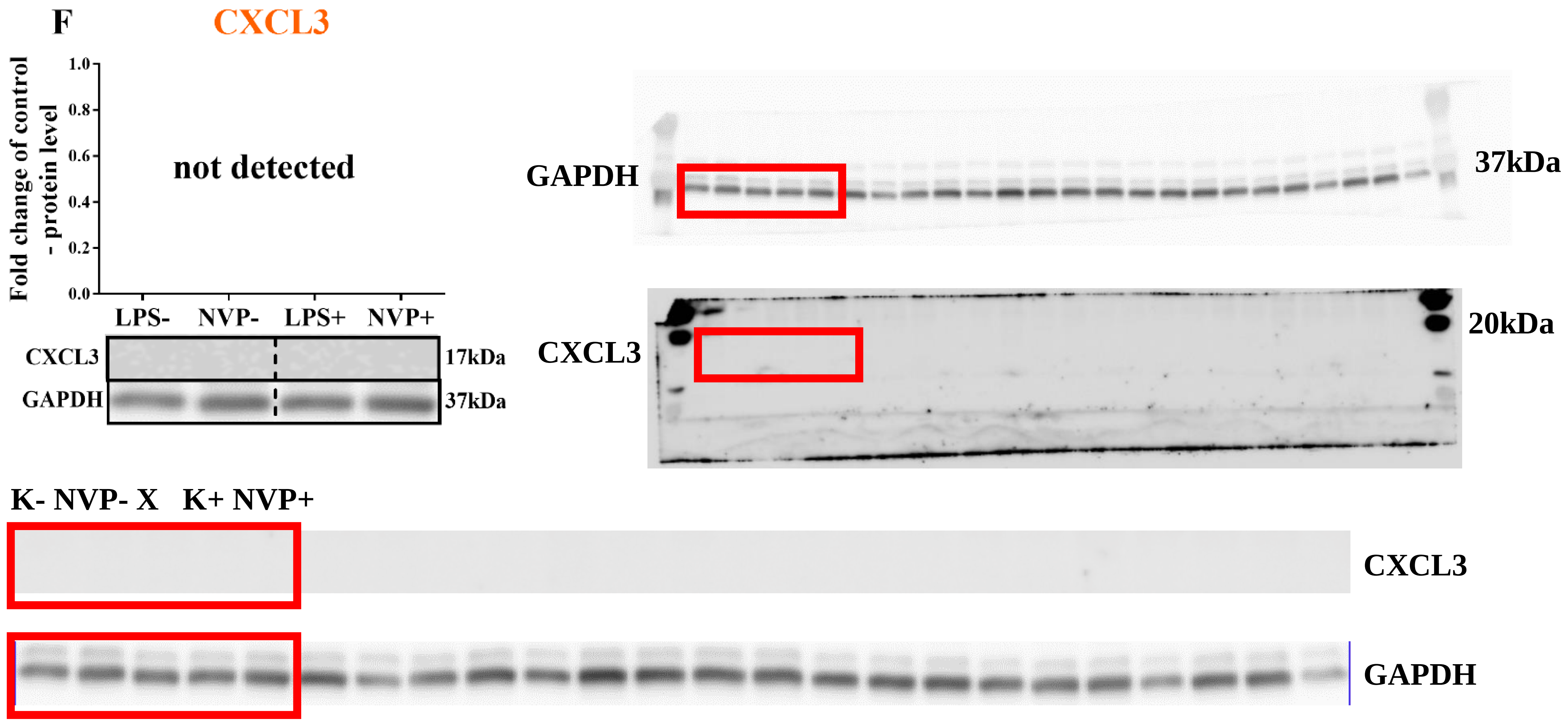

key symbols:

K-/LPS- – vehicle-treated non-stimulated cells ; NVP- - NVP-treated non-stimulated cells

K+/LPS+ – LPS-stimulated cells; NVP+ - NVP-treated LPS-stimulated cells

X - another compound
